# Low temperature thermal RAFT depolymerization: the effect of Z-group substituents on molecular weight control and yield†

**DOI:** 10.1039/d4sc07518h

**Published:** 2025-01-22

**Authors:** Nethmi De Alwis Watuthanthrige, Anastasiia Moskalenko, Asja A. Kroeger, Michelle L. Coote, Nghia P. Truong, Athina Anastasaki

**Affiliations:** a Laboratory of Polymeric Materials, Department of Materials, ETH Zurich Vladimir Prelog Weg 5 8093 Zurich Switzerland athina.anastasaki@mat.ethz.ch; b Institute for Nanoscale Science and Technology, College of Science and Engineering, Flinders University Bedford Park South Australia 5042 Australia

## Abstract

The labile end-groups inherent to many controlled radical polymerization methodologies, including atom transfer radical polymerization (ATRP) and reversible addition–fragmentation chain-transfer (RAFT) polymerization, can trigger the efficient chemical recycling of polymethacrylates yielding high percentages of pristine monomer. Yet, current thermal solution ATRP and RAFT depolymerization strategies require relatively high temperatures (*i.e.* 120–170 °C) to proceed, with slower depolymerization rates, and moderate yields often reported under milder reaction conditions (*i.e.* lower temperatures). In this work, we seek to promote the low temperature RAFT depolymerization of polymethacrylates *via* regulating the Z-group substitution of dithiobenzoate. While electron-withdrawing *meta* and *para* substituents, including trifluoromethyl (CF_3_) and trifluoromethoxy (OCF_3_), compromised the percentage of monomer recovery at 90 °C (*e.g.* 18% of conversion), instead the incorporation of electron-donating groups in the benzene ring, such as methoxy (OMe) and tertiary butoxy (OtBu), had a remarkable effect leading to up to four times higher conversions (*e.g.* 75%). Notably, electron-withdrawing Z-groups imposed control over depolymerization, reflected in the gradual decrease of the molecular weight during the reaction, as opposed to electron-donating groups which underwent a more uncontrolled depolymerization pathway. Density Functional Theory (DFT) calculations revealed accelerated bond fragmentation for electron-donating Z-groups, further supporting our findings. Taken altogether, this work highlights the importance of RAFT agent selection to either lower the reaction's temperature while maintaining high conversions, or induce control over the depolymerization.

## Abstract 2

Labile RAFT end-groups are crucial for poly(methacrylate) depolymerization. Electron-donating Z-groups improve monomer recovery at lower temperatures, while electron-withdrawing groups provide molecular weight control during depolymerization.

## Graphical abstract

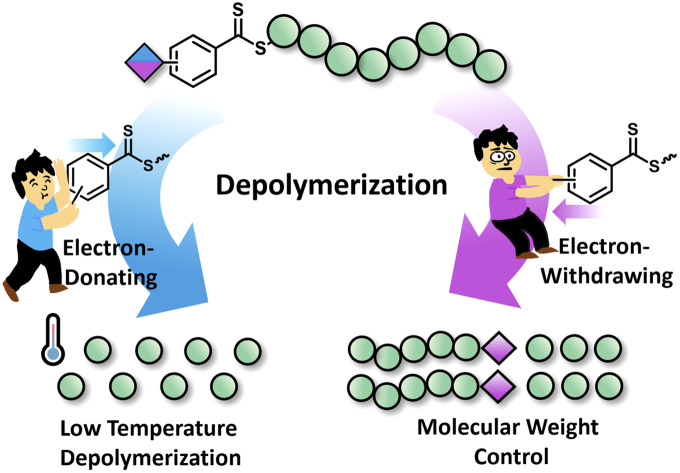

## Introduction

Chemical recycling offers an attractive proposition of breaking down polymers and converting them back to their constituent monomers.^1,2^ One of the most dominant and industrially relevant chemical approaches to depolymerize polymeric materials is the so-called pyrolysis, a process that employs extreme temperatures (>400 °C) to induce breakage, often accompanied by side reactions and impure recovered monomer.^3,4^ To induce lower temperature depolymerization reactions, polymers synthesized by reversible deactivation radical polymerization (RDRP), also referred to as controlled radical polymerization (CRP), have recently been explored.^5^ State-of-the-art polymerization methodologies, such as atom transfer radical polymerization (ATRP) and reversible addition–fragmentation chain-transfer (RAFT) polymerization, can inherently install labile end-groups at the overwhelming majority of the produced polymer chains, thus enabling efficient cleavage under milder reaction conditions.^6–10^

While bulk depolymerizations of RDRP polymers still require high temperatures to operate (*i.e.* 220 °C),^11–14^ solution depolymerizations can proceed at lower temperatures by leveraging polymer dilution.^3,5^ However, even in solution depolymerization, ATRP systems typically require 170 °C to effectively proceed.^15–17^ For example, Matyjaszewski and co-workers have utilized either copper or iron catalysis to depolymerize a range of chlorine-terminated polymethacrylates at 170 °C, yielding up to 76% of retrieved monomer.^16,18^ The chemical recycling of bromine-terminated polymers was recently demonstrated by our group *via* favouring initiation and depropagation over lactonization, also reaching high monomer yields.^19^ However, when the depolymerization of ATRP polymers was attempted at lower temperatures, significantly lower reaction rates and conversions were obtained. For instance, Ouchi and co-workers only regenerated 24% of monomer at 120 °C when the reaction was left to proceed overnight (*i.e.* 24 h),^20^ and these results were consistent with Matyjaszewski's work which also revealed compromised yields at these lower temperatures.^17^ To boost the depolymerization conversion under milder reaction conditions, light was recently explored as an external stimulus.^21,22^ In the presence of visible light irradiation and concurrent with moderate heating (*i.e.* 120 °C), higher conversions could be achieved (*i.e.* ∼70%), although at even lower temperatures (*i.e.* 100 °C), the final conversion could not surpass 5% when low catalyst loadings were employed.^21^ As such, purely thermal ATRP depolymerization systems do not currently regenerate high amounts of monomer at lower temperatures.

An analogous situation can also be observed in thermal RAFT depolymerizations.^23–25^ While exciting work from the Gramlich group highlighted the propensity of bottlebrush-like materials to efficiently depolymerize at lower temperatures (*i.e.* 70 °C),^26^ for non-bulky analogues (*e.g.* polymethylmethacrylate), 120 °C appears to be the ideal temperature to maximize the depolymerization yield, which has been reported to be up to 86%.^24^ While equally high conversions can be reached under photothermal conditions (*i.e.* concurrent light irradiation and heat at 100 °C), as reported by the Sumerlin group and our group independently, exclusively thermal depolymerizations at 100 °C led to significantly compromised conversions.^27–29^ For example, when either trithiocarbonates or dithiobenzoates were used as the RAFT agents, the resulting polymers could only undergo up to 40% of monomer regeneration when subjected to the optimized depolymerization conditions.^27^ Taken altogether, these findings highlight the inability of both RAFT and ATRP systems to induce an efficient depolymerization reaction at lower temperatures.

**Fig. 1:**
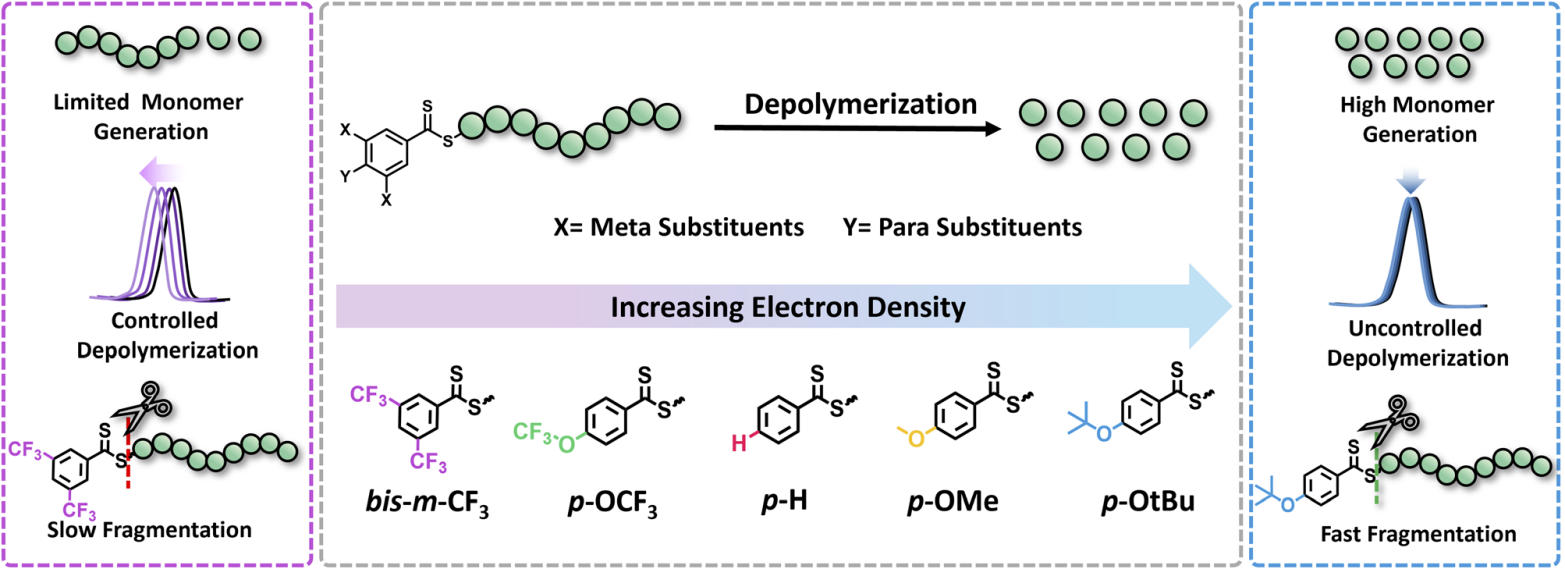
Conceptual diagram illustrating the electronic modulation of raft agents *via* substitution at *para* and *meta* positions of the dithiobenzoates and their effect on depolymerization in terms of monomer generation, control and fragmentation efficiency.

In this work, we seek to examine the impact of electronic effects of RAFT agents on thermal RAFT depolymerization by modulating the activity of the Z-group. While such an effect has been widely recognized and understood in the respective polymerizations,^30–33^ it has not yet been leveraged to enhance depolymerizations. RAFT agents with both electron-donating (ED) and electron-withdrawing (EW) substituents will be synthesized including both *para* and *meta* substituents in the Z-groups of the RAFT agents, such as trifluoromethyl (CF_3_), trifluoromethoxy (OCF_3_), hydrogen (H), methyl (Me), methoxy (OMe), and tertiary-butoxy (OtBu), while taking into account their Hammett constant values (Fig. 1). The propensity of these CTAs to trigger lower temperature depolymerization will be thoroughly examined *via* detailed kinetics. In parallel, the possibility of the various substituents to trigger either a controlled or an uncontrolled depolymerization will also be explored. Last but not least, DFT calculations will be employed to confirm the experimental findings.

## Results and discussion

**Fig. 2:**
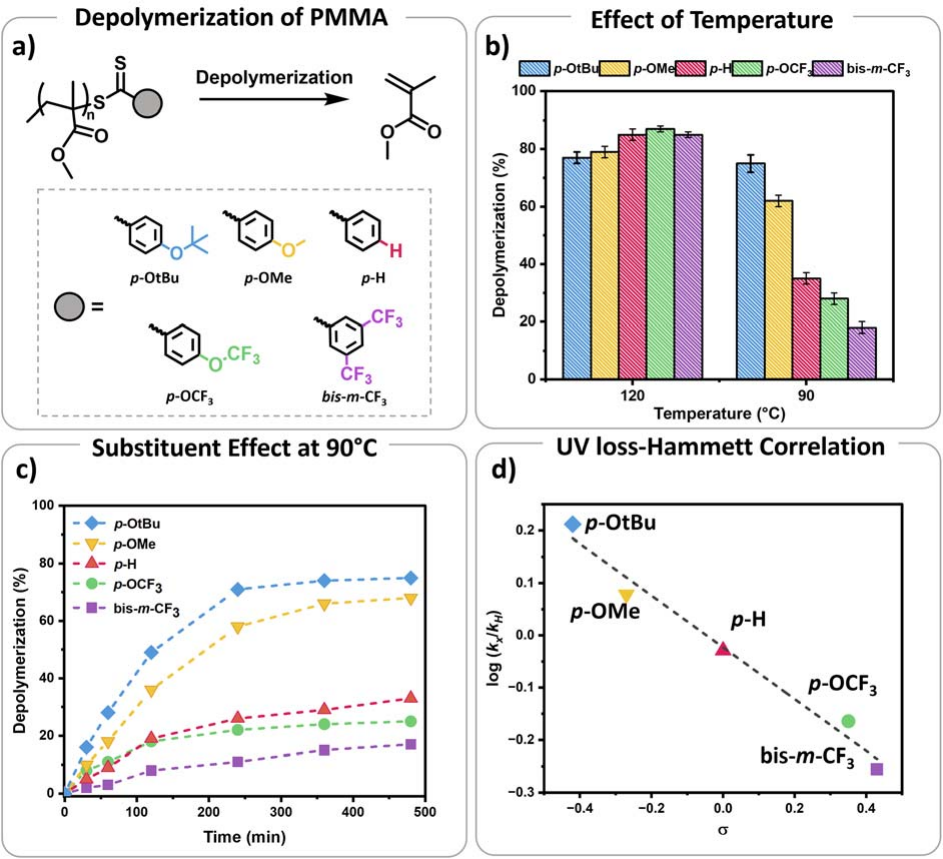
(a) Schematic representation of depolymerization conditions of PMMA RAFT polymers with different substituents (*p*-OtBu, *p*-OMe, *p*-H, *p*-OCF_3_, bis-*m*-CF_3_), conditions: 5 mM PMMA repeat unit concentration in dioxane, (b) final depolymerization conversions of different substituents at 120 °C and 90 °C, (c) depolymerization kinetics of polymers with different substituents at 90 °C, (d). Hammett plot for the rate of UV signal loss of the polymers with different substituents plotted against their Hammett substituent constants (*σ*) 
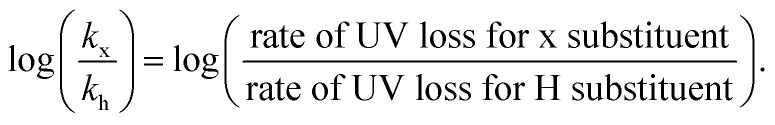

To initiate our study, a series of CTAs were synthesized with variable Z-group substituents as indicated in Fig. 1. Polymerization literature clearly suggested that Z-groups primarily influence the reactivity of C=S bond towards radicals, with *para* and *meta* substitution resulting in polymers with lower dispersity and higher end-group fidelity.^34^ In contrast, *ortho* substitutions including *ortho*–*para* disubstitution, disrupt the conjugation between the phenyl and C=S bond, reducing polymerization control.^35^ In terms of the electronic properties of CTAs, EW groups provide superior control in the initial stages of polymerization,^34–36^ while ED substituents exhibit faster kinetics in photo-iniferter processes.^37^ Considering these observations (kinetics, and chain transfer coefficients (*C*_tr_)), as well as synthetic feasibility, we carefully selected substituents with Hammett constants ranging from −0.42 to 0.45, including four *para* substituents (*p*-OtBu, *p*-OMe, *p*-H, *p*-OCF_3_) and a *meta* substituent (bis-*m*-CF_3_) (Fig. S1 and S2†).^38,39^ Thermal RAFT polymerization was then employed to polymerize methyl methacrylate (MMA) under judiciously optimized conditions yielding PMMAs with various end-groups (Fig. 2a). To aid an accurate comparison between these polymers, comparable degrees of polymerization (DP) and dispersities were targeted, resulting in polymers with narrow molar mass distributions (*Đ* ≤ 1.18) and controlled molecular weights (*M*_n_ = 7200–7500, Fig. S3–S5 and Table S1†). In addition, all synthesized polymers exhibited similar livingness, based on the amount of the radical initiator concentration utilized (*e.g.* ∼98%),^40^ thus indicating high end-group fidelity (Table S1†). Thermal depolymerizations were conducted at 5 mM repeat unit concentration, using dioxane as the solvent following our previously established literature protocol.^24,25^ At 120 °C, all polymers, irrespective of whether they possessed an ED or EW substituent, displayed comparable depolymerization yields ranging from 79% to 87% (Fig. 2b and Table S2†). This finding suggests that the electron density of the Z-group does not significantly affect the depolymerization extent at 120 °C. We therefore envisaged that lower reaction temperatures would better highlight potential differences between the different substituents. To investigate this further, multiple depolymerizations were performed for all the substituents, at lower temperatures (70 °C, 80 °C, 90 °C, 100 °C, and 110 °C; Table S2†).

At slightly lower temperatures (*i.e.* 110 °C), no major change in either the depolymerization rate or the final depolymerization yield was observed as all polymers could be efficiently depolymerized, resulting between 81% and 87% of retrieved monomers (Table S2†). However, a different observation was witnessed at 100 °C. In particular, the unsubstituted analogue (*p*-H) underwent up to 62% depolymerization. Instead, EW substituents only reached 55% and 50% of recovered monomer for the *p*-OCF_3_ and bis-*m*-CF_3_ Z-group respectively. Notably, both ED substituents, namely *p*-OtBu, and *p*-OMe, generated much higher percentages of monomer with the final depolymerization yields recorded at 81% and 75% respectively. The differences between EW and ED substituents became even more pronounced at 90 °C. While the most ED substituent, *p*-OtBu, maintained an overall high final depolymerization conversion (*i.e.* 75%), the most EW substituent, bis-*m*-CF_3_ only reached 18% of conversion. As a control experiment, the unsubstituted analogue (*p*-H) depolymerized to intermediate values (*i.e.* 35%). Following this trend, when the depolymerizations were conducted at even lower temperatures (*e.g.* 80 °C), a very low percentage of recovered monomer was obtained for the unsubstituted Z-group (*p*-H; *i.e.* 13%), while no meaningful depolymerization could be observed for the EW substituent (*i.e.* 2%). In contrast, the ED donating groups yielded up to 45% of monomers. It is noted that even lower temperatures (*i.e.* 70 °C) resulted in a further decrease in the overall monomer yields, suggesting that the thermodynamic limit of the system had been reached, although the electronic effects still can be observed.

Considering these observations, we were eager to gain further insights by investigating the kinetic profile of the polymers bearing different Z-group substituents at 90 °C (Fig. 1c). Within the first two hours, the *p*-OtBu substituent displayed 49% depolymerization, compared to 36% for *p*-OMe, 19% for *p*-H, 18% for *p*-OCF_3_ and only 8% for bis-*m*-CF_3_. After 4 h, polymers with *p*-OtBu and *p*-OMe substituents depolymerized up to 71% and 58% respectively, while polymers with more EW substituents showed less than 30% of depolymerization (Table S3†). Initial size exclusion chromatography (SEC) analysis using a UV detector revealed that polymers with ED Z-groups rapidly lost their end-groups during depolymerization (Fig. S6 and Table S3†), unlike those with EW Z-groups. This observation is reasonable as higher monomer recovery is intrinsically related to higher percentages of end-group activation, subsequently leading to end-group loss. While dithiobenzoate end-group loss in RAFT polymers typically occurs *via* thermolysis or amination,^41–44^ at this temperature the loss of end-group is primarily attributed to activation by dioxane-derived radicals.^45^ This activation results in the formation of solvent-derived dithiobenzoate small molecules, as shown in our previous work.

A Hammett plot was constructed by plotting the normalized UV loss rates (relative to *p*-H) against the corresponding substituent constants (*σ*) to gain further insights into end-group activation (Fig. S7,† and 2d). A negative slope (*ρ* < 0) was revealed at 90 °C, indicating that the end-group activation is favoured by high electron density. This points to a mechanism where the rate determining step likely involves an electrophilic intermediate or transition state, which benefits from increased electron density around the aromatic ring.^6,7^ Importantly, we observed that starting from 120 °C, the end-group activation reactions transitioned from being less sensitive (*ρ* ≈ 0) to more sensitive (*ρ* < 0) to the ED substituents in the Z-groups as the temperature decreased (Fig. S8†). While it is known that the slope of the Hammett plot can vary depending on temperature and solvent,^46^ we hypothesize that this shift in sensitivity is primarily due to increased radical generation from the solvent at higher temperatures (120 °C). At these higher temperatures, the initiation step is mainly driven by the rapid rate of radical formation, diminishing the influence of the substituents. In contrast, at lower temperatures, where solvent radical formation is slower, the reaction becomes more sensitive to the substituents. As such, the observed lower temperature depolymerization of RAFT polymers was attributed to the enhanced electron density effects and under exclusively thermal conditions we were able to not only reach, but also surpass the conversions achieved with previously reported photothermal methodologies.^27–29,47^

**Fig. 3:**
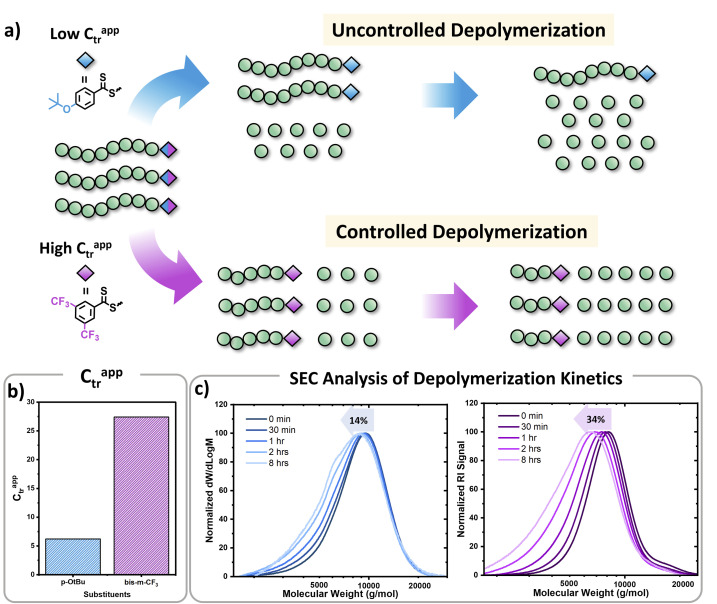
(a) Schematic representation of the concept for uncontrolled and controlled depolymerization, (b) *C*_tr_s for CTAs containing *p*-OtBu and bis-*m*-CF_3_ Z-groups, (c) SEC traces during the depolymerization of the PMMA polymers containing *p*-OtBu Z-group (blue traces, left) and bis-*m*-CF_3_ Z-group (purple traces, right) (conditions: 2.5 mM repeat unit concentrations, 100 °C in dioxane).

In parallel, we were interested in probing the potential of each substituent to mediate either an uncontrolled or a controlled depolymerization. Uncontrolled depolymerization is defined as the process by which, upon chain-end activation, the instant unzipping of the polymer chain takes place, resulting in the complete release of all monomers.^5^ In other words, chains unzip fully-one at a time while the molecular weight remains relatively constant throughout the reaction.^48^ Instead, in a perfectly controlled depolymerization, all polymer chains are simultaneously activated and unzip uniformly as long as deactivation dominates over depropagation (Fig. 3a). In such situations, the original molecular weight of the polymer gradually decreases, resulting in a notable shift in *M*_n_ towards lower values. So far, there is only one single report of controlled depolymerization whereby an excess amount of CTA (*i.e.* dithiobenzoate) was employed to favour deactivation over depropagation.^48^ Considering the difference in electronic properties between EW and ED groups we envisioned that, especially at the lower temperatures employed (*i.e.* 90 and 100 °C), EW Z-groups would potentially lead to a more controlled depolymerization (Fig. S9†). In particular, according to the polymer literature, EW Z-groups exhibit enhanced control owing to their presumably higher *C*_tr_.^35^ To verify this, the *C*_tr_ values for each RAFT agent were experimentally determined following well established literature protocols (Fig. S10†).^49^ As expected, ED substituents displayed lower *C*_tr_ values (*i.e.* 6.2 (*p*-OtBu), 6.8 (*p*-OMe)), while the EW substituents showed much higher values (*i.e.* 10 (*p*-OCF_3_) and 27.4 (bis-*m*-CF_3_)). The *C*_tr_ of the unsubstituted one was calculated to be at 7.1. The stark contrast in *C*_tr_ between *p*-OtBu and bis-*m*-CF_3_ (Fig. 3b) is expected to lead from an uncontrolled to a more controlled depolymerization pathway. As indicated in Fig. 3c, detailed kinetic analysis revealed that for *p*-OtBu, a mere overall shift of just 14% in *M*_n_ could be recorded by SEC when 79% of monomer was generated. In more detail, at 17% of monomer regeneration an *M*_n_ shift (defined as the percentage decrease in *M*_n_ compared to the original *M*_n_ (*M*_n,0_) of the polymer: *M*_n_ shift = ((*M*_n,0_ − *M*_n,t_)/*M*_n,0_) × 100%) of just 4% was observed, while at 33% of conversion, only 9.6% of *M*_n_ shift was attained. The final *M*_n_ shift of 14% was recorded at 59% of conversion while at even higher conversions, no further *M*_n_ shift could be seen. The uncontrolled nature of this reaction is attributed to the ED nature of the substituent leading to rapid depropagation, and poor deactivation during the depolymerization, as depicted in Fig. 3a (blue), resulting in a relatively constant molar mass distribution throughout the reaction. In contrast, polymers with EW Z-groups, such as bis-*m*-CF_3_, demonstrated a vastly different kinetic profile. Specifically, an *M*_n_ shift of 7.5% could already be noticed at 13% of monomer regeneration, and this value was further increased to 15.5, 26 and 31% when the conversion reached 23, 44, and 62% respectively. A final *M*_n_ shift of 34% could be obtained at 68% conversion, as indicated by SEC (Table S4†). This gradual decrease in molecular weight is attributed to enhanced deactivation, which facilitates a more controlled depolymerization pathway, where monomers unzip one by one (Fig. 3a (purple)). While in our previous work, a 20-fold excess of the CTA was required to impose control over the depolymerization,^48^ here the presence of intact CTAs as end-groups in the polymer alone is sufficient to achieve the same level of control, owing to their significantly higher *C*_tr_.

**Fig. 4:**
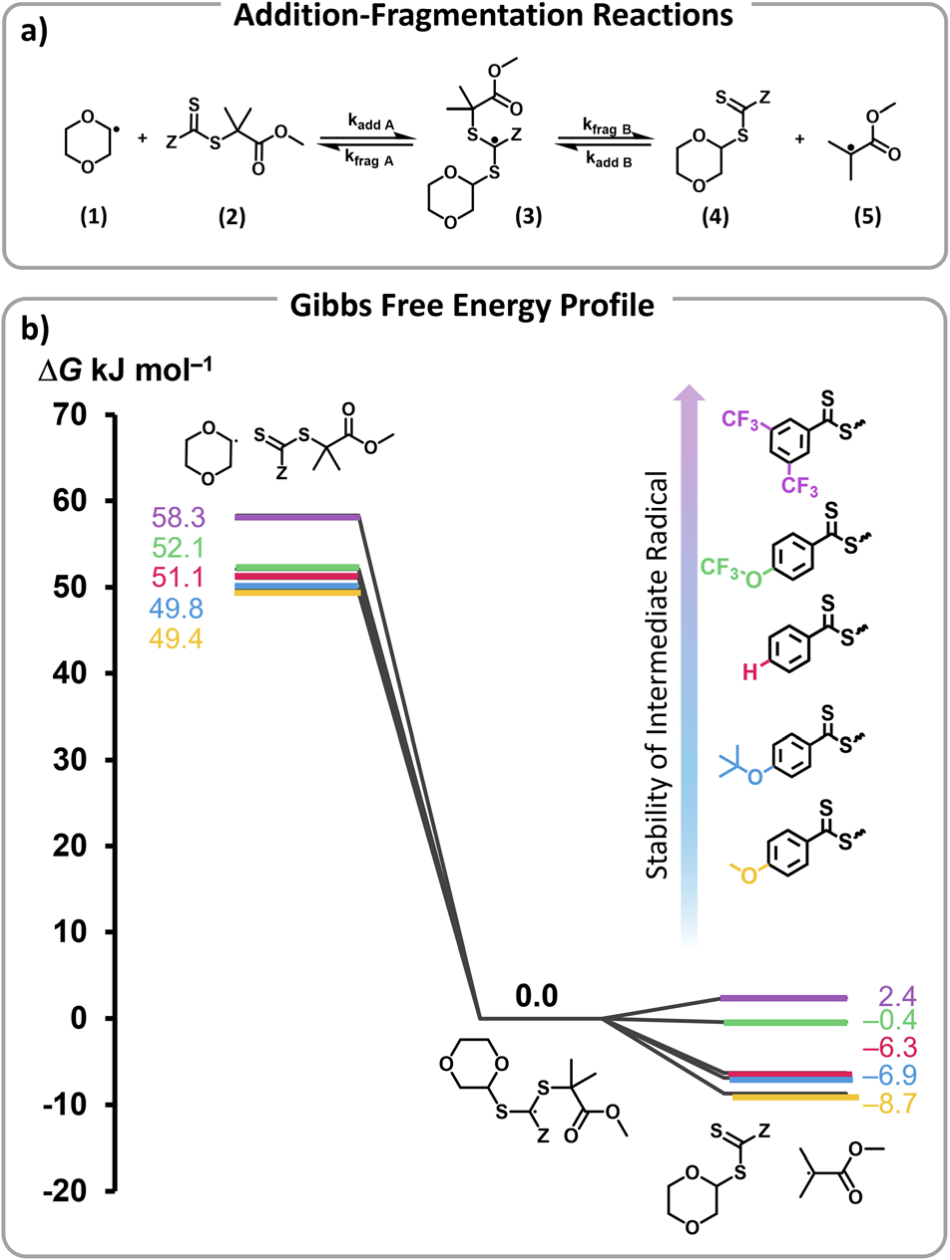
(a) Model initiation reaction studied. A solvent derived dioxane radical (1) undergoes radical addition to the RAFT end-group of the polymer (2) yielding the RAFT intermediate radical (3) which then undergoes fragmentation to afford a solvent derived RAFT agent (4) and a methacrylate derived radical (5), which can then undergo depolymerization. (b) Gibbs free energy profile (90 °C, 1,4-dioxane) for the addition–fragmentation reaction in (a) with different Z-group substituents.

To rationalize the efficient depolymerizations induced by ED Z-groups, and the controlled nature of the reaction with EW Z-groups, density functional theory (DFT) calculations were performed. In a previous study, we demonstrated that solvent-derived radicals are primarily responsible for initiating depolymerization.^45^ In particular, trace peroxide radicals in 1,4-dioxane were hypothesized to form dioxane radicals upon heating (Scheme S1a†). These radicals add to RAFT end-groups, activating chain ends and generating radicals that can then undergo depropagation to regenerate monomer (Scheme S1b†).^45^ The negative slope of the Hammett plot for Z-group substituents supports the formation of an electrophilic intermediate, likely a radical, which is stabilized by the electron-rich RAFT end-group, highlighting electronic effects on the mechanism. Building on this understanding, our modelling approach specifically examined the addition of dioxane radicals (1) to the respective modelled RAFT agent with a single unit of MMA (2), which through an intermediate radical (3) subsequently fragments into a solvent-derived RAFT agent (4) and a methyl methacrylate-derived radical (5) (Fig. 4a).^45^

The Gibbs free energy profiles at 90 °C in 1,4-dioxane for the different substituents (Fig. 4b) showed that the methyl methacrylate-derived radical (5) is a significantly better leaving group than the dioxane-derived radical (1), thus enhancing the overall efficiency of the transfer reaction across all substituents. However, at lower temperatures, such as 90 °C, the calculated Gibbs free fragmentation energies (
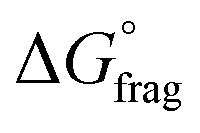
, Fig. 4b and Table S5†) indicate that fragmentation of the intermediate radical (3) is slightly thermodynamically disfavoured for the bis-*m*-CF_3_ Z-group 
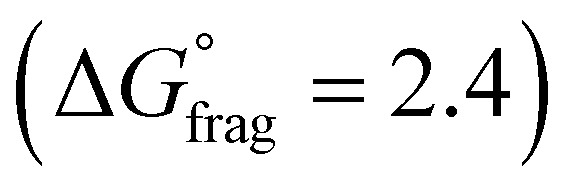
. For the *p*-OCF_3_ Z-group 
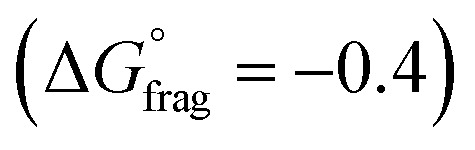
, fragmentation is nearly thermoneutral, while for the other Z-groups, it is only mildly thermodynamically favored 
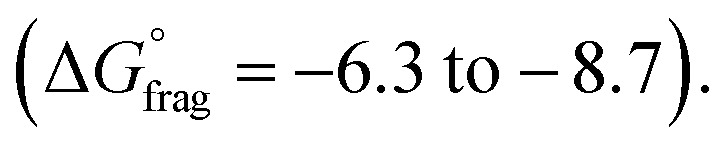
 This leads to rate retardation, particularly for bis-*m*-CF_3_ and *p*-OCF_3_, due to the persistence of the radical intermediate (3), increasing termination and reducing the overall depolymerization efficiency, in line with our experimental findings. While the calculations focussed on thermodynamic factors, kinetics are expected to follow similar trends for these types of reactions.^50^

Consistent with previous studies, increasing EW character of the Z-group reduces the stability of the RAFT agent, while ED substituents enhance stability by allowing greater resonance stabilization, which is otherwise inhibited by EW groups.^50^ This consequently reduces the thermodynamic favourability of fragmentation, prolonging the lifetime of the RAFT-adduct radical and increasing possibilities for termination or deactivation events. This phenomenon aligns very well with the observed reduction of depolymerization extents, which arise from increased termination, and the substantial shifts toward lower molecular weights due to enhanced deactivation in polymers containing EW Z-groups. Additionally, steric hindrance plays a significant role in termination events during depolymerization. For instance, less hindered Z-groups (*e.g.*: Z = *p*-H) lead to more frequent termination events than ED Z-groups (*p*-OtBu and *p*-OMe), even under favourable thermal conditions. Conversely, bulky groups like *p*-OtBu prevent termination of the RAFT intermediate (3), resulting in greater depolymerization efficiency than *p*-OMe, which is theoretically more favourable for fragmentation (Fig. 4b, 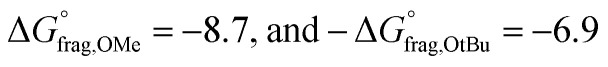). As temperature increases, fragmentation becomes more thermodynamically favourable, enhancing depolymerization efficiency and minimizing sensitivity to the substituents' electronic properties.

## Conclusions

In conclusion, we demonstrated the critical role of RAFT agents on the depolymerization of poly(methyl methacrylate) at a 5 mM concentration in dioxane. At higher temperatures (120 °C), the reaction was less sensitive to Z-group effects. However, at lower temperatures (90 °C) the reaction effectively displayed sensitivity towards the substituents, and this was further confirmed by Hammett analysis. Notably, depolymerization reached 75% at 90 °C, and 45% at 80 °C, particularly with ED groups like *p*-OtBu. Additionally, EW Z-groups displayed 34% *M*_n_ shift during depolymerization, indicating greater deactivation and more controlled monomer release resulting in uniform polymer chain shortening. DFT calculations supported these observations, showing that the electronic structure of RAFT end-groups significantly influences the favourability of the addition and fragmentation reactions at 90 °C. ED groups facilitated faster fragmentation of the intermediate radical, enhancing depolymerization at lower temperatures, whereas EW groups hindered fragmentation by making the intermediate radical persistent. At elevated temperatures, fragmentation became more thermodynamically favourable, reducing sensitivity to electronic effects. These findings suggest that tuning the electronic properties of the RAFT end-group can optimize depolymerization efficiency, yield, and control, opening new possibilities for various opportunities.

## Data availability

All the data supporting this article have been included in the main text and the ESI.†

## Author contributions

N. D. A. W. – conceptualization, methodology, synthesis, investigation, writing, reviewing and editing. A. M. – synthesis, investigation, data analysis A. A. K. − DFT calculations, writing, reviewing and editing. M. L. C. – DFT calculation, reviewing and editing. N. P. T. – conceptualization, writing, reviewing and editing. A. A. – conceptualization, project administration, supervision, funding acquisition, writing, reviewing and editing.

## Conflicts of interest

The authors declare no conflicts of interest.

## Supplementary Material

SC-016-D4SC07518H-s001
