# Supplementary material for: Low temperature thermal RAFT depolymerization: the effect of Z-group substituents on molecular weight control and yield
Source: Chem Sci. 2025 Jan 22;16(8):3516–22. doi: 10.1039/d4sc07518h (PMC11774200; doi:10.1039/d4sc07518h)
Supplement: SC-016-D4SC07518H-s001 [file SC-016-D4SC07518H-s001.pdf]

Supplementary Information (SI) for Chemical Science.
This journal is © The Royal Society of Chemistry 2025

Low Temperature Thermal RAFT Depolymerization; the Effect of Z-group Substituents on Molecular Weight Control and Yield

Nethmi De Alwis Watuthanthrige[a], Anastasiia Moskalenko[a], Asja A. Kroeger[b], Michelle L. Coote[b], Nghia Truong[a], Athina Anastasaki*[a]

[a] Laboratory of Polymeric Materials, Department of Materials, ETH Zurich, Vladimir-Prelog-Weg 5, Zurich 8093, Switzerland

[b] Institute for Nanoscale Science and Technology, College of Science and Engineering, Flinders University, Bedford Park, South Australia 5042, Australia

Contents

1. Methods ... 4

1.1. Materials. ... 4

1.2. NMR spectroscopy ... 4

1.3. Size-exclusion chromatography (SEC). ... 4

1.4 Mass Spectrometry (MS) ... 4

1.5. Synthesis of CTAs ... 4

1.5.1. 2-cyanopropan-2-yl 4-methoxybenzodithioate (*p*-OMe CTA) ... 4

1.5.2. 2-cyanopropan-2-yl 4-(tert-butoxy)benzodithioate (*p*-OtBu CTA) ... 5

1.5.3. 2-cyanopropan-2-yl 3,5-bis(trifluoromethyl)benzodithioate (bis-*m*-CF_3 CTA) ... 5

1.5.4. 2-cyanopropan-2-yl 4-(trifluoromethoxy)benzodithioate (*p*-OCF_3) ... 6

1.6. Synthesis of PMMA ... 6

1.6.1. typical Polymerization procedure of MMA using 2-cyanopropan-2-yl benzodithioate (*p*-H) as the RAFT agent. ... 6

1.7 Chain Transfer Coefficients (C_{tr}) Calculation ... 7

1.8. Depolymerization of PMMA ... 8

1.8.1. Typical Depolymerization procedure of PMMA with 5mM solution ... 8

1.9. Constructing Hammet Plot ... 8

2. Supporting Data ... 10

3. Total energies ... 18

3.1. Overall fragmentation processes ... 18

4. Cartesian coordinates ... 20

4.1 Structures used to evaluated addition-fragmentation reactions ... 21

5. References ... 33

1. Methods

1.1. Materials.

All materials were purchased from either Sigma Aldrich or Fischer Scientific unless otherwise stated. Monomers were filtered through basic alumina before use.

1.2. NMR spectroscopy.

^{1}H-NMR spectra were recorded on a Bruker Avance-300 spectrometer using $CDCl_3$ as the NMR solvent.

1.3. Size-exclusion chromatography (SEC).

SEC was measured on a Shimadzu equipment comprising a CBM-20A system controller, LC-20AD pump, SIL-20A automatic injector, 10.0 μm bead-size guard column (50 x 7.5 mm) followed by three KF-805L columns (300 x 8 mm, bead size: 10 μm, pore size maximum: 5000 Å), SPD-20A ultraviolet (UV) detector, and an RID-20A differential refractive index (RI) detector. For the UV measurements, 310 nm wavelength was selected. The column temperature was maintained at 40 °C using a CTO-20A oven. The flow rate was set to 1 ml/min and with N, N-dimethylacetamide (DMAc, Acros, HPLC grade, with 0.03 w/v LiBr) as the eluent. Molecular weights were determined relative poly(methyl methacrylate) standards with molecular weights ranging from 5,000 to 1.5 x 106 g/mol (Agilent Technologies). All SEC samples were dissolved in DMAc and passed through 0.45 μm filters prior to analysis.

1.4 Mass Spectrometry (MS)

Mass spectrometry (MS) experiments were either performed on a Bruker Maxis I quadrupole-time-of-flight (QTOF) at 4500V emitter voltage with electrospray ionization (ESI) as ion source and direct injection or on a Thermo Scientific QExactive GC Orbitrap (GC-MS) with Ion-Trap (OrbiTrap) and electron impact (EI) as ion source.

1.5. Synthesis of CTAs

1.5.1. 2-cyanopropan-2-yl 4-methoxybenzodithioate (*p*-OMe CTA)

The synthesis procedure was adopted from existing literature.[1]

A mixture of Mg (0.25 g) in dry THF (40 mL), along with a pinch of iodine, was deoxygenated for 20 minutes in a 150 mL two neck round bottom flask equipped with a condenser and a magnetic stirrer bar. A solution of 4-bromoanisole (1.25 mL, 10 mmol) in 10 mL of dry THF was added to

the deoxygenated system while purging it with nitrogen. The reaction mixture was heated gently to initiate the reaction and stirred for 3 h. After 3 hours, carbon disulfide (1 mL, 16.7 mmol) was added dropwise to the solution and stirred for a further 3 hrs. Excess solvent was removed under vacuum, and the resulting deep red viscous liquid was dissolved in 0.1 M K_2CO_3 solution (100 mL). The dissolved red color solvent was then filtered and washed with ethyl ether (2 x 50 mL) to remove unreacted materials and dissolved $I_{2,}$ and poured into a flask equipped with a magnetic stirrer. An aqueous solution of 1 N iodine (2 g of KI and 1.27 g of I_2 crystals in 10 mL of distilled water) was added to the reaction mixture until the solution started to change color from dark red to pink as the formed disulfide precipitated. To quench the excess $I_{2,}$ a few crystals of $Na_2S_2O_3$ were added. The resultant aqueous solution was extracted with methylene chloride and dried over anhydrous sodium sulfate. The excess solvent was evaporated under vacuum to obtain the disulfide bridge compound as a red solid.

The resultant bridge compound (0.65 g, 1.8 mmol) and AIBN (0.34 g, 2.1 mmol) were dissolved in 120 mL of ethyl acetate in a 250 mL two neck round bottom flask equipped with a condenser and a magnetic stirrer. The solution was degassed for 20 minutes and refluxed (100 °C) under nitrogen atmosphere for 24 hrs. The reaction was monitored with TLC and once all the bridge compounds converted to product the reaction was stopped, and the excess solvent was removed under vacuum. The crude product was purified by a flash chromatography (hexane/ethyl ether=7:3) obtaining reddish pink color crystalline solid (yield 86%, purity 99.3%). ^{1}H NMR (300 MHz, CDCl3) δ 7.93 (d, J = 9.0 Hz, 2H), 6.81 (d, J = 9.0 Hz, 2H), 3.80 (s, 3H), 1.87 (s, 6H).

1.5.2. 2-cyanopropan-2-yl 4-(tert-butoxy)benzodithioate (*p*-OtBu CTA)

The synthesis of novel *p*-OtBu CTA was conducted following the established procedure for *p*-OMe CTA. The reaction used 1-bromo-4-(tert-butoxy)benzene as the starting material, in equimolar quantities. The product obtained was a reddish-pink crystalline solid with an 80% yield (99.3% purity). ^{1}H NMR (300 MHz, CDCl3) δ 7.86 (d, J = 8.8 Hz, 2H), 6.89 (d, J = 8.9 Hz, 2H), 1.87 (s, 6H), 1.35 (s, 12H), ^{13}C NMR (126 MHz, CDCl3) δ 220.98, 161.03, 138.99, 128.04, 122.02, 120.24, 79.94, 41.58, 28.95, 26.66, m/z= 293.0903.

1.5.3. 2-cyanopropan-2-yl 3,5-bis(trifluoromethyl)benzodithioate (bis-*m*-CF_3 CTA)

The synthesis was carried out using a published procedure in literature.[2]

A 25% solution of sodium methoxide in methanol (4.57 mL, 20mmol) was added to a suspension of sulfur (0.64 g, 20 mmol)and 3,5-bis(trifluoromethyl)benzyl bromide (1.83 mL, 10 mmol) in methanol (50mL) in a round-bottom flask equipped with a condenser and magnetic stirring. After 3 hrs, the solvent was removed and then resulting dark brown crude product was dissolved in a diluted solution of $NaHCO_3$. After being washed with ethyl ether (2× 50 mL), an aqueous solution of iodine 0.96 N (10.42 mL, 10 mmol) was added dropwise. During the addition, the solution started to change color from dark brown to pink as the disulfide precipitated. After elimination of excess I_2 with few crystals of $Na_2S_2O_3$, the mixture was extracted with ethyl ether and dried over sodium sulfate. After removal of the solvent, the crude product (0.87 g) was not purified because of its instability toward cleaning procedures and was directly used for the subsequent reaction.

The reaction with AIBN and bridge compound was carried out using same procedure as described in synthesis of Cyanopropyl-2-yl(4-methoxy) Dithiobenzoate (*p*-OMe CTA) using crude disulfide bridged compound (0.87 g, 1.2 mmol) and AIBN (0.81 g, 1.9 mmol) after a flash chromatography (hexane/ethyl ether=7:3) as a reddish pink color crystalline solid (yield 58%, purity 99.3%). ^1H NMR (300 MHz, $CDCl_3$) δ 8.23 (s, 2H), 7.98 (s, 1H), 1.90 (s, 6H).

1.5.4. 2-cyanopropan-2-yl 4-(trifluoromethoxy)benzodithioate (*p*-OCF_3)

The synthesis of *p*-OCF_3 CTA was conducted following the established procedure for *p*-OCF_3 CTA.[1] The reaction used 1-bromo-4-(trifluoromethoxy)benzene as the starting material in equimolar quantities, as reported in the literature. The product obtained was a reddish-pink crystalline solid with a 65% yield (98.5% purity). ^1H NMR (300 MHz, $CDCl_3$) δ 7.90 (d, J = 8.9 Hz, 1H), 7.16 (d, J = 8.4 Hz, 2H), 1.88 (s, 4H).

1.6. Synthesis of PMMA

1.6.1. typical Polymerization procedure of MMA using 2-cyanopropan-2-yl benzodithioate (*p*-H) as the RAFT agent.

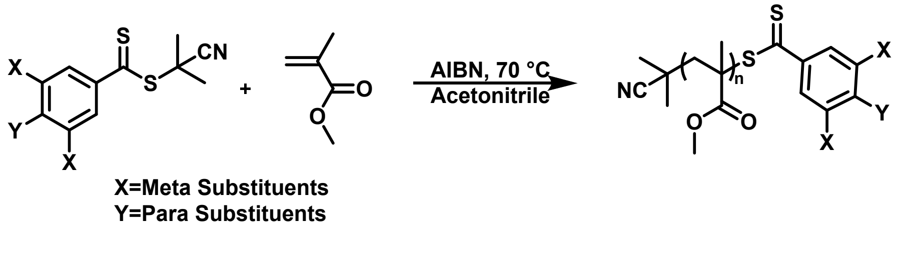

A typical polymerization procedure targeting a DP of 75 is described here with *p*-H CTA and methyl methacrylate (MMA). In a 25 mL round-bottom flask, 63.2 mg of 2-cyanopropan-2-yl benzodithioate (285.4 μmol, 1 equiv) and 4.68 mg of AIBN (28.5 μmol, 0.1 equiv) were dissolved in 4 mL of acetonitrile. Then, 4 mL of MMA (39.9 mmol, 140 equiv) and a magnetic stir bar were added. The flask was sealed with a septum and deoxygenated by bubbling nitrogen gas for 15 minutes.

Polymerization was conducted in an oil bath at 70 °C with stirring at 400 rpm. Periodic samples were collected under a nitrogen blanket for ^{1}H-NMR analysis and were filtered through a 0.45 μm PTFE syringe filter before SEC analysis. The polymerization was terminated at 40% monomer conversion by removing the flask from the oil bath and unsealing the septum.

The same protocol was applied for PMMA synthesis using other RAFT agents, adjusting the amounts of CTA based on their molecular weights.

1.7 Chain Transfer Coefficients (C_{tr}) Calculation

C_{tr} was calculated using a modified version of the method developed by Moad, Rizzardo, and Thang.[3]

Into a 25 mL round bottom flask, 63.2 mg of 2-cyanopropan-2-yl benzodithioate (285.4 μmol, 1 equiv) and 4.68 mg AIBN (28.5 μmol, 0.1 equiv) were dissolved in 4 mL acetonitrile. Subsequently, 4 mL of MMA (39.9 mmol, 140 equiv) and a stirrer bar were added, and the flask was sealed with a septum, prior to deoxygenation by nitrogen bubbling for 15 min. Polymerization was conducted in an oil bath at 70 °C with a 400-rpm stirring rate. 0.1 mL samples were taken periodically under a nitrogen blanket for ^{1}H-NMR and SEC analysis. For SEC samples, 50 µL samples were diluted with 1.5 mL DMAc and passed through a syringe filter (0.45 µm PTFE membrane).

The conversion of the monomer was calculated through NMR by integrating the vinyl peaks with respect to methoxy peak (polymer +monomer) around 3.5 ppm. The conversion of the RAFT agent was calculated by the intensity of the RAFT agent UV signal in SEC, and the apparent C_{tr} was calculated by the equation $C_{tr} = \frac{d(\ln [RAFT\ agent])}{d(\ln [MMA])}$. Thus the slope of a ln[RAFT agent] vs ln[MMA] plot is the apparent C_{tr}.

1.8. Depolymerization of PMMA

1.8.1. Typical Depolymerization procedure of PMMA with 5mM solution

In a 250 mL Schlenk flask, 20 mg of PMMA was dissolved in 40 mL of 1,4-dioxane. To this, 15 mg of poly(ethylene glycol) monomethyl ether (molar mass 350 g/mol) was added as an internal standard for ^{1}H NMR analysis. The flask was then sealed with a rubber septum and deoxygenated by bubbling nitrogen gas for 20 minutes.

The flask was placed in an oil bath preheated to one of the target temperatures (70 °C, 80 °C, 90 °C, 100 °C, or 120 °C) to initiate the reaction. It was submerged that the solution inside was approximately 2 cm below the surface of the oil bath.

To take samples, the flask was periodically removed from the oil bath and quickly cooled in a water bath to room temperature. Samples were then collected under a nitrogen blanket. For SEC analysis, approximately 3 mL of the sample solution was evaporated, dissolved in 1.5 mL of DMAc, and filtered through a 0.45 μm PTFE syringe filter.

1.9. Constructing Hammet Plot

The UV signal loss during each depolymerization was calculated using Equation S1 and subsequently plotted against depolymerization time to track the kinetics. The rate constant for UV signal loss, $k_{\text{uv-loss}}$, was determined from these kinetic plots. To facilitate comparisons, the UV loss rates of other polymers were normalized to that of the unsubstituted CTA-containing polymer. These normalized rates were then plotted against the Hammett substituent constant for each substituent group (Equation S2). The slope of this plot, denoted as the reaction rate constant ρ, is influenced by the substituent effect and depends on both the type and position of each substituent within the molecule. The constant ρ is specific to the reaction and is also affected by conditions such as temperature and solvent. (Typical values for ρ in common reactions range from about −1to +1)

1. Larger ρ values (whether +ve or -ve): reaction is more sensitive to substituent effects, indicating that the electronic nature of the substituent has a stronger influence on the reaction rate
2. Smaller ρ values : reduced sensitivity, indicating that the substituent effect is weaker in the reaction mechanism.

3. +ve ρ value: the reaction rate increases with electron-withdrawing substituents and reaction mechanism involves a buildup of negative charge on the transition state or intermediate, which is stabilized by electron-withdrawing groups.
4. -ve ρ value: the reaction rate increases with electron-donating substituents and buildup of positive charge or electrophilic state in the transition state or intermediate, which is stabilized by electron-donating groups.
5. $\rho=0$, no sensitivity to substituents, and no charge is built or lost.

These relations can be developed to elucidate the mechanism of a reaction. As the value of ρ is related to the charge during the rate determining step, mechanisms can be devised based on this information.

$$UV\ Loss = \frac{(UV_{polymer,0} - UV_{polymer,t})}{UV_{polymer,0}}$$ **Equation S 1**

Where $UV_{polymer,0}$ is the UV signal of the polymer at time 0, $UV_{polymer,t}$ is the UV signal of the polymer at time t.

$$\frac{logk_x}{logk_H} = \sigma\rho$$ **Equation S 2**

Where k_x is the rate of UV-loss in polymers with substituent x, k_H is the rate of UV-loss in polymers with substituent H, σ is the Hammett substituent constant (available in literature),[4, 5] and ρ is the overall reaction rate constant.

1.10 Computational Methods

Geometry optimizations and frequency calculations were performed at the SMD[6]-(1,4-dioxane)-M062X[7]/6-31G(d)[8-11] level of theory. For evaluation of the addition-fragmentation equilibria, thorough conformational searches (i.e., with resolutions of 120° around sp^3-sp^3 bonds and 180° around sp^2-sp^3 bonds) were carried out to obtain the global minimum conformation of each structure of interest. For calculations of the descriptors, equivalent conformers of reference compounds were used as recommended in ref. [12] In all cases, more accurate electronic energies were subsequently calculated at the SMD[6]-(1,4-dioxane)-wB97X-D[13]/aug-cc-pVTZ[14, 15] level of

theory. Solution phase Gibbs free energies were calculated via the “direct method” of Truhlar and co-workers,[16] in which ideal gas partition functions, evaluated here under the harmonic oscillator / rigid rotor approximation, were applied directly to the solution-phase geometries and frequencies. A correction for the change of state from 1 atm to 1 M was included.[17] All geometry optimizations, frequency, and single point energy calculations were carried out using the Gaussian16 program package.[18] 3D visualizations were generated with CYLview.[19]

2. Supporting Data

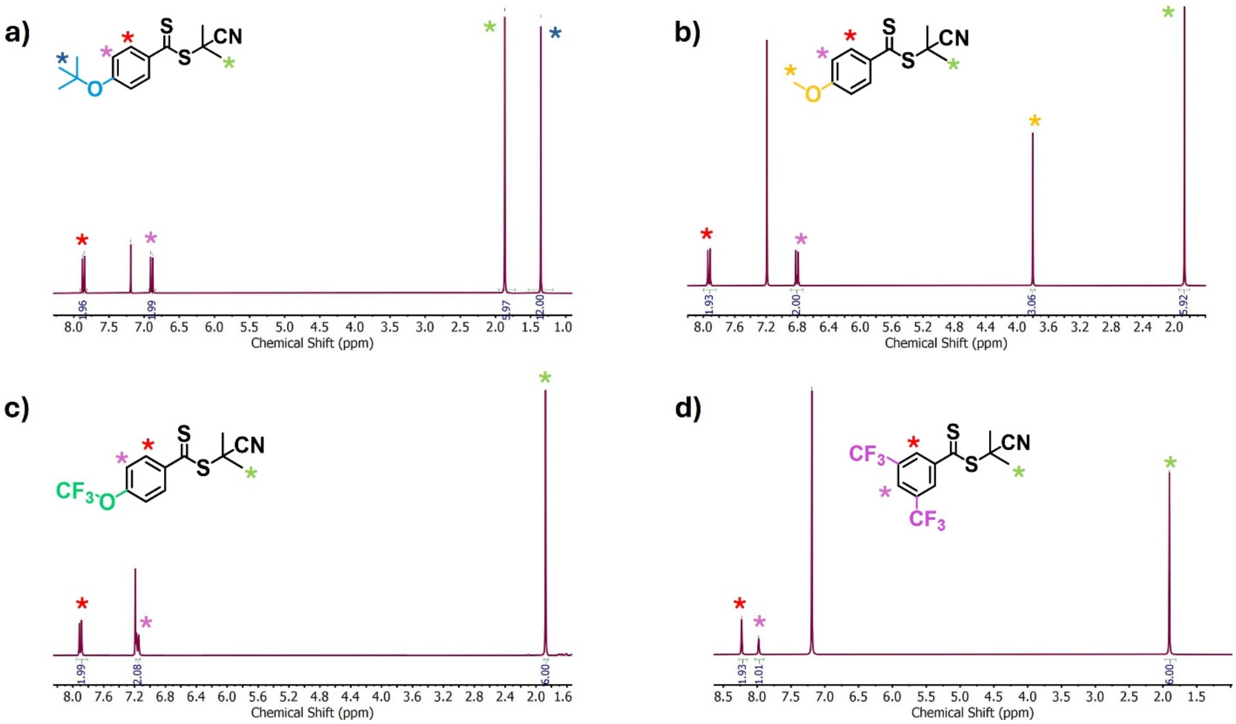

Figure S 1: ^{1}H NMR spectra of the synthesized CTAs a) *p*-OtBu, b) *p*-OMe, c) *p*-OCF$_3$, d) *bis*-*m*-CF$_3$

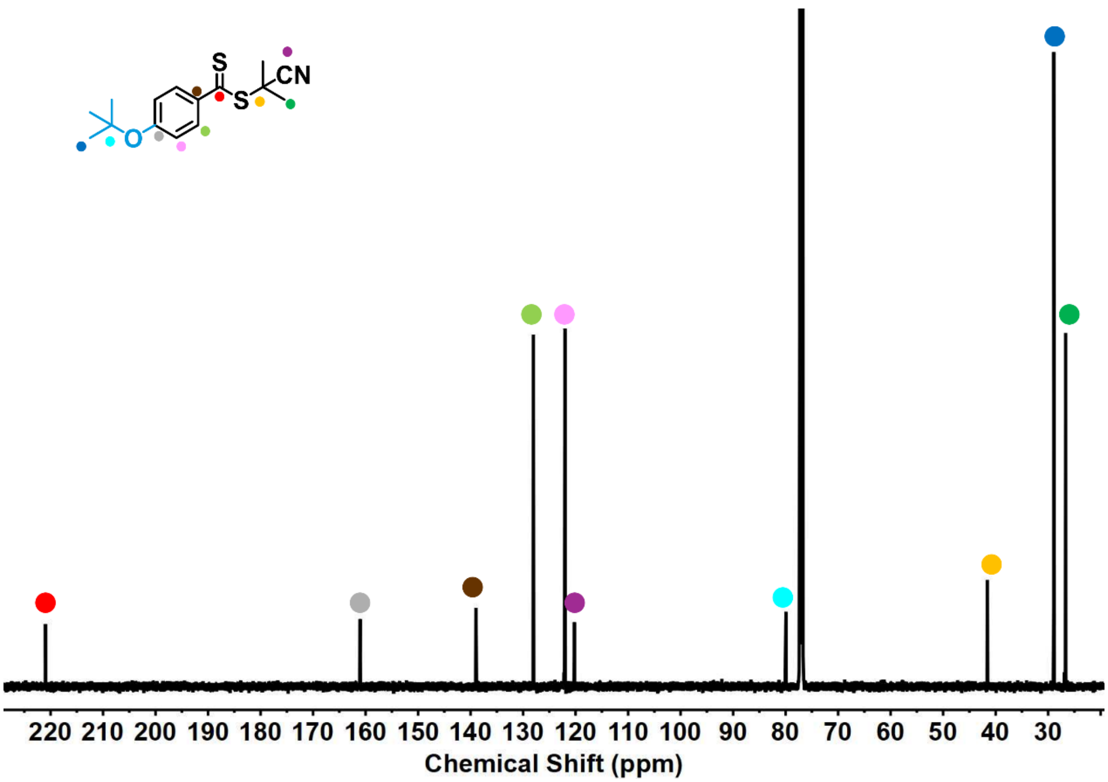

Figure S 2: ^{13}C NMR spectrum of the novel *p*-OtBu CTA

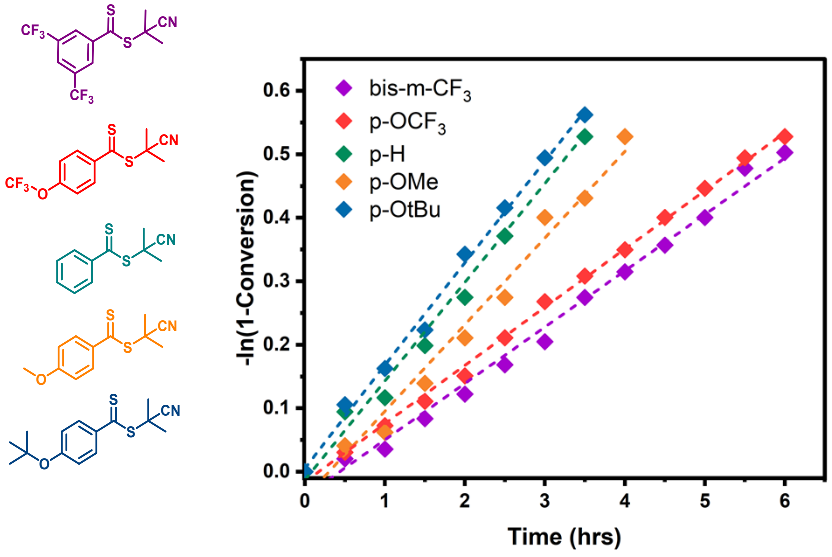

Figure S 3: Polymerization Kinetics; semi-logarithmic plots for the polymerization of methyl methacrylate at 70 °C. AIBN: DTB-X:MMA=0.1:1:140 (X=*p*-OtBu, *p*-OMe, *p*-H, *p*-OCF$_3$, bis-*m*-CF$_3$)

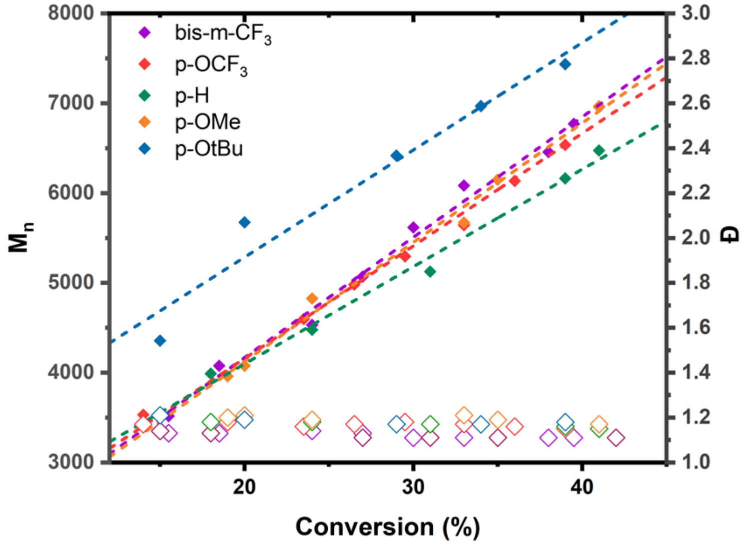

Figure S 4: M_n and *Đ* evolution for the polymerization of methyl methacrylate at 70 °C. AIBN: DTB-X:MMA=0.1:1:140 (X=*p*-OtBu, *p*-OMe, *p*-H, *p*-OCF$_3$, bis-*m*-CF$_3$)

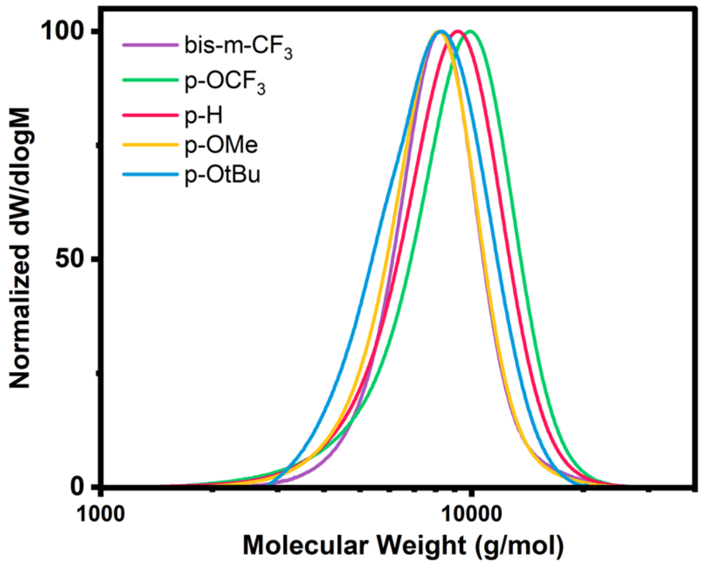

Figure S 5: SEC traces of DP75 PMMA polymers with bis-*m*-CF$_3$, *p*-OCF$_3$, *p*-H, *p*-OMe, *p*-OtBu Z-groups

Table S 1: $M_{n(exp)}$, Dispersity (Đ) values obtained through SEC traces, Calculated livingness of the polymers with DTB-bis-*m*-CF_3, DTB-*p*-OCF_3, DTB-*p*-H, DTB-*p*-OMe, DTB-*p*-OtBu Z-groups

Polymer	M_n	Đ	Livingness (%)[a]
Bis-*m*-CF_3-$PMMA_{75}$	7200	1.11	97.6
p-OCF_3-$PMMA_{75}$	7300	1.11	97.6
p-H-$PMMA_{75}$	7500	1.15	98.3
p-OMe-$PMMA_{75}$	7300	1.17	98.2
p-OtBu-$PMMA_{75}$	7500	1.18	98.3

[a] livingness was calculated using $L = \frac{[CTA]_0}{[CTA]_0 + 2f[I_0](1 - e^{-k_d t})(1 - \frac{f_c}{2})}$ equation[20]

where $[CTA]_0$ and $[I]_0$ are the initial concentrations of chain transfer agent and initiator, respectively. The term "2" means that one molecule of initiator gives two primary radicals with a certain efficiency f (typically 0.5 for diazo initiators). The term $1 - f_c/2$ represents the number of chains produced in a radical–radical termination event with f_c the coupling factor ($f_c = 1$ means 100% bimolecular termination by combination; $f_c = 0$ means 100% bimolecular termination by disproportionation).

Table S 2: Depolymerization conversions at different temperatures for PMMA DP 75 polymers with different substituents ranging from 120 °C to 70 °C

Substituent	Depolymerization Conversion (%)[a]					
	120 °C	110 °C	100 °C	90 °C	80 °C	70 °C
p-OtBu	79	82	81	75	45	16
p-OMe	79	81	75	62	35	12
p-H	85	86	62	35	13	1
p-OCF_3	87	87	55	28	8	2
Bis-*m*-CF_3	85	85	50	18	2	0

[a] the depolymerization conversions were calculated using the average of at least 3 experimental measurements.

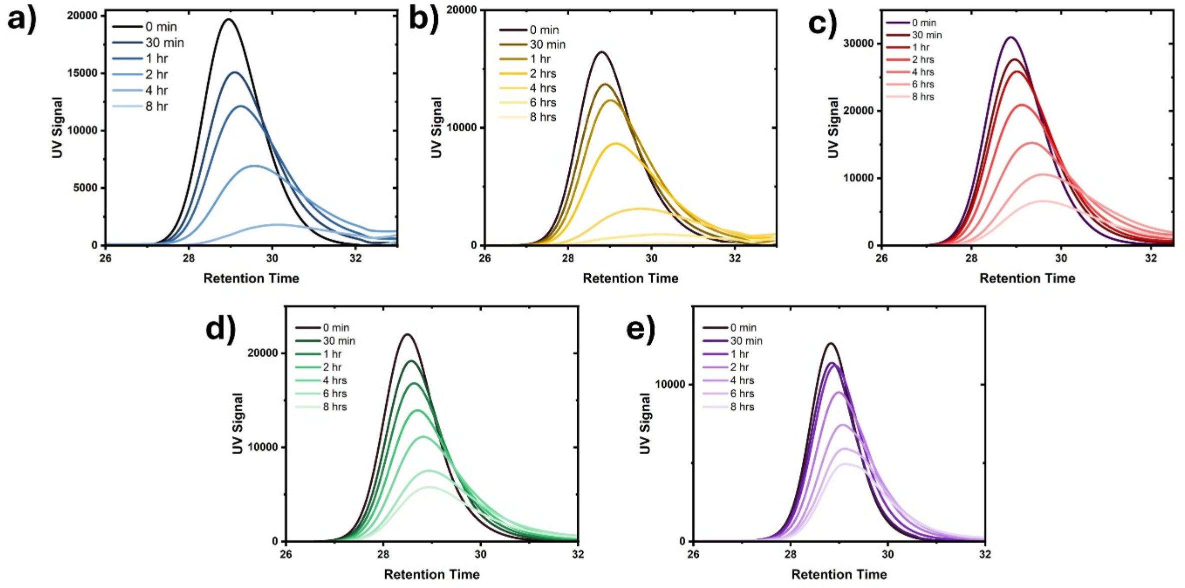

Figure S 6: UV signal variations in the SEC UV detector during the kinetic experiments for polymers with different substituents at 90 °C, a) *p*-OtBu, b) *p*-OMe, c) *p*-H, d) *p*-OCF_3 and e) *bis*-*m*-CF_3

Table S 3: UV loss (%) and depolymerization conversions (%) at 90 °C for polymers with different substituents (conditions: 5mM repeating unit concentration, in Dioxane)

Time (mins)	*p*-OtBu		*p*-OMe		*p*-H		*p*-OCF3		Bis-*m*-CF3	
	UV Loss (%)	Depol (%)	UV Loss (%)	Depol (%)	UV Loss (%)	Depol (%)	UV Loss (%)	Depol (%)	UV Loss (%)	Depol (%)
0	0	0	0	0	0	0	0	0	0	0
30	12	16	9	10	4	5	6	8	4	2
60	20	28	10	18	7	9	9	11	10	3
120	43	49	28	36	18	19	16	18	12	8
240	84	71	66	58	42	26	31	22	26	11
360	97	74	89	66	72	29	61	24	35	15
480	99	75	99	68	84	33	78	25	44	17

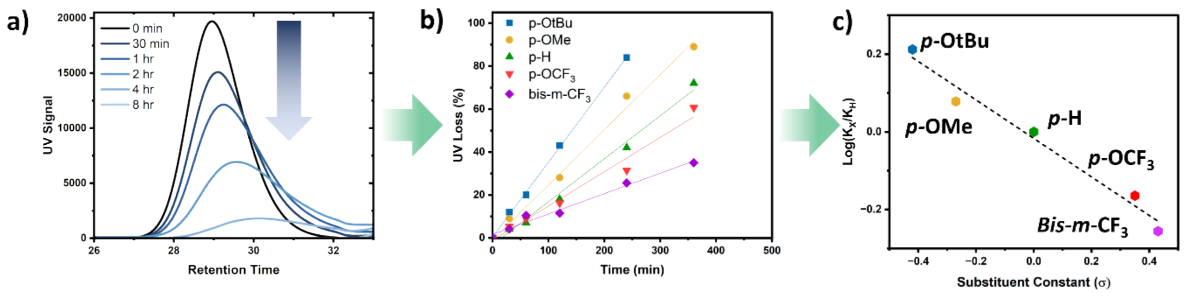

Figure S 7: Steps for constructing Hammett plots: (a) Performing SEC-UV analysis and calculate the percentage of UV loss using Equation S1; (b) determining the UV-loss rate for each polymer with different substituents; (c) normalize the rates relative to the UV-loss rate of the non-substituted polymer and construct the Hammett plot by plotting the normalized logarithmic rate ($\log(k_x/k_H)$) against the substituent constants (σ).

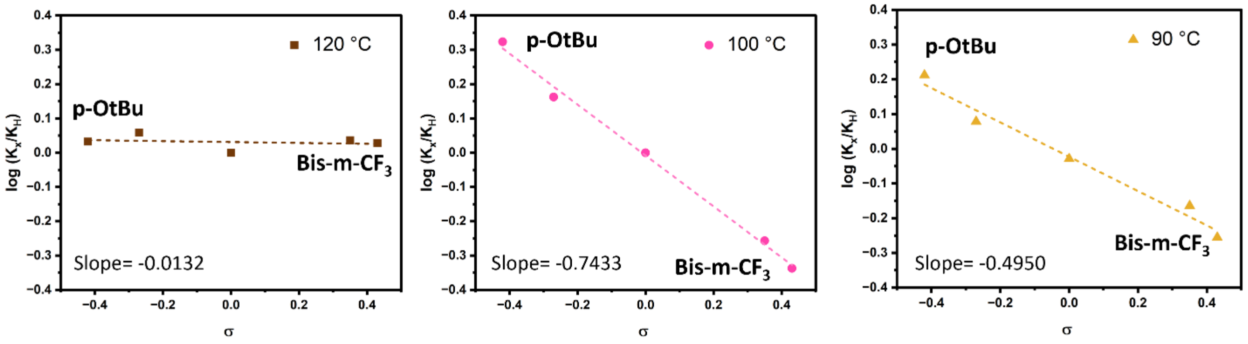

Figure S 8: Variations in Hammette plots a) at 120 °C, b) at 100 °C, and c) at 90°C

Table S 4: Depolymerization conversions (%) and M_n Shift (%) values at 100 °C for *p*-OtBu and bis-*m*-CF_3 containing polymers (conditions: 2.5 mM repeating unit concentration, in Dioxane)

Time	*p*-OtBu		Bis-*m*-CF_3	
	Depol (%)	M_n Shift (%)*	Depol (%)	M_n Shift (%)*
0	0	0	0	0
30	17	3.8	13	7.5
60	33	9.6	23	15.5
120	59	14	44	26
240	75	14	62	31
360	79	14	68	34

* M_n Shift= $\frac{(M_{n,0} - M_{n,t})}{M_{n,o}} \times 100\%$ where $M_{n,0}$ is the M_n of the polymer at time 0, and $M_{n,t}$ is the M_n of the polymer at time t

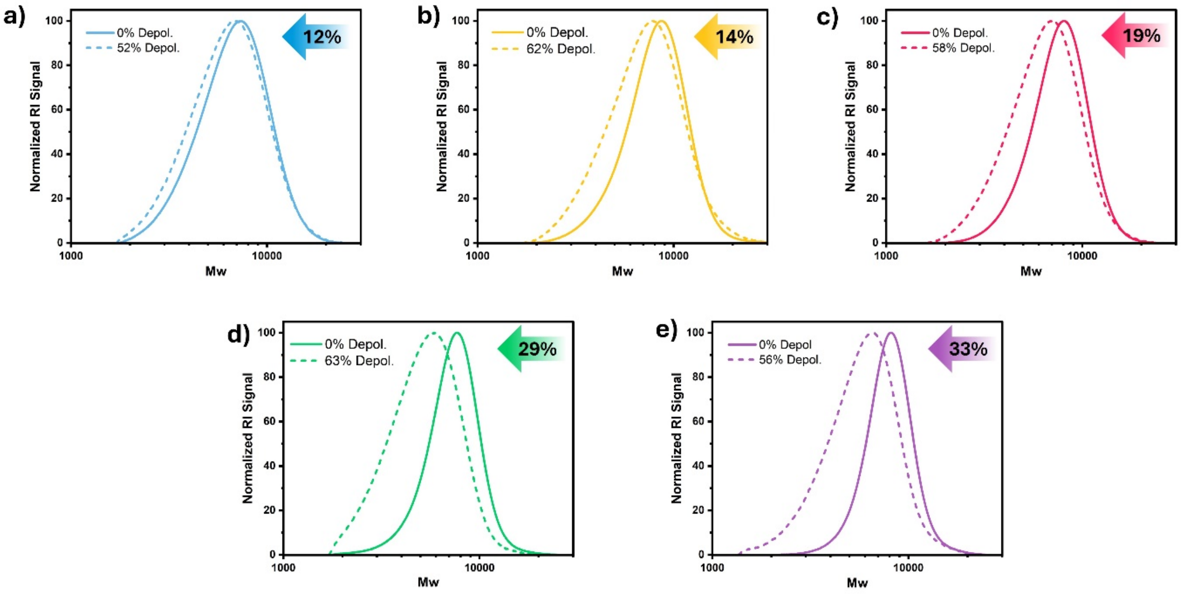

Figure S 9: M_n Shift of PMMA polymers containing a) *p*-OtBu, b) *p*-Ome, c) *p*-H, d) *p*-OCF$_3$, and e) bis-*m*-CF$_3$ Z groups at similar conversions during depoloymerization at 100 °C. (5 mM concenteation, dioxane as the solvent)

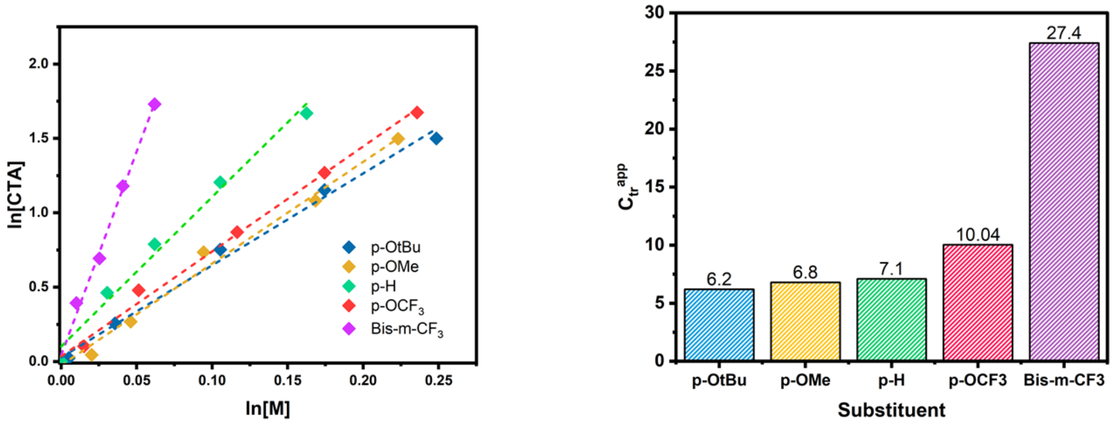

Figure S 10: The CTA concentration vs monomer concentration plotted through the kinetics experiments(left), Calculated Ctr values for polymers with bis-*m*-CF$_3$, *p*-OCF$_3$, *p*-H, *p*-OMe, *p*-OtBu Z- groups (right)

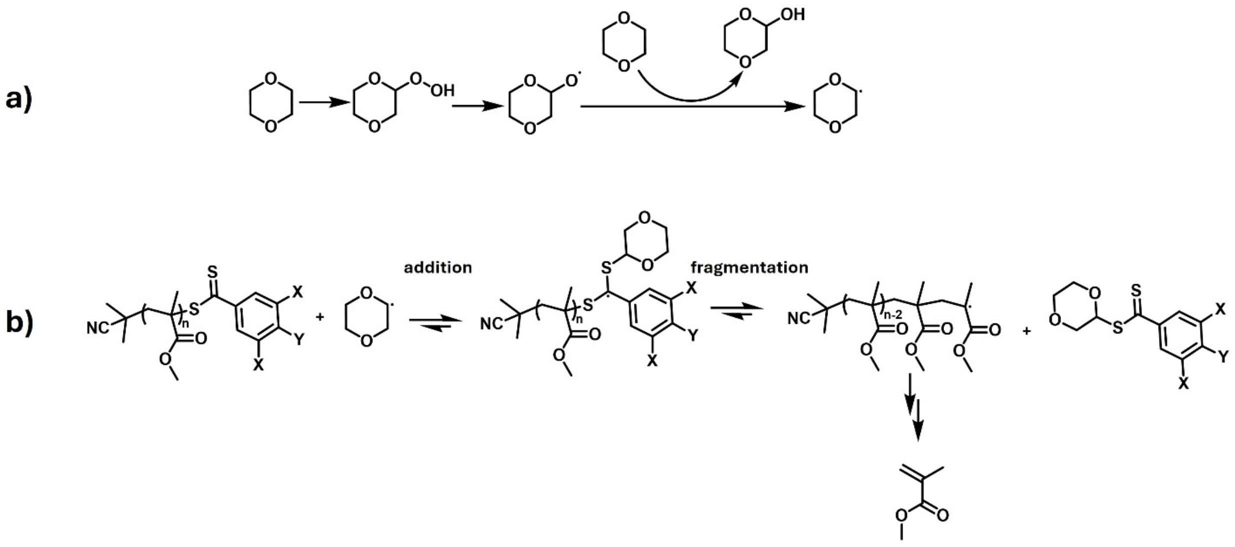

Scheme S 1: (a) Proposed mechanism leading to 1,4-dioxanyl radical. (b) Proposed mechanism of solvent-initiated thermal RAFT depolymerization in 1,4-dioxane through dioxane radical pathway

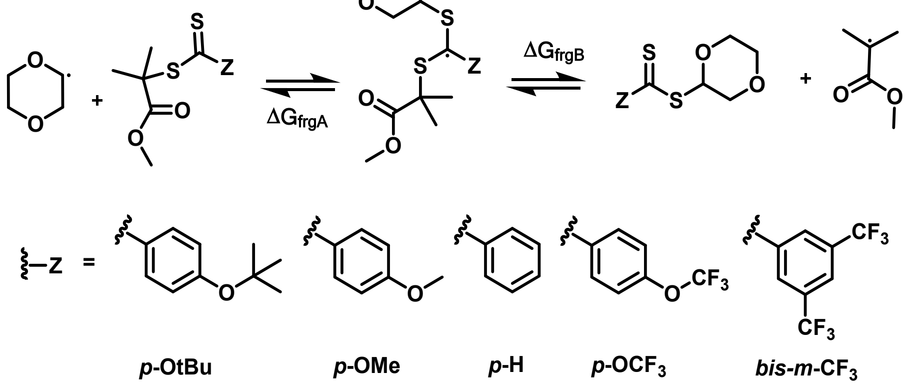

Scheme S 2: Model addition-fragmentation reactions studied.

Table S 5: Gibbs free fragmentation energies (kJ mol^{-1}, 1,4-dioxane) calculated relative to the adduct radical in both directions.[a]

		Temp in °C		
		90	100	120
p-OMe	forming dioxane radical	49.4	48.0	45.3
	forming MMA radical	-8.7	-10.4	-13.8
p-OtBu	forming dioxane radical	49.8	48.4	45.6
	forming MMA radical	-6.9	-8.5	-11.9
p-H	forming dioxane radical	51.1	49.8	47.0
	forming MMA radical	-6.3	-8.0	-11.4

p-OCF_3	forming dioxane radical	52.1	50.6	47.8
	forming MMA radical	-0.4	-2.1	-5.4
bis-*m*-CF_3	forming dioxane radical	58.3	56.7	53.4
	forming MMA radical	2.4	0.5	-3.4

[a] Calculated at the wB97X-D/aug-cc-pVTZ//M062X/6-31G(d) level of theory using the SMD solvent model.

3. Total energies

3.1. Overall fragmentation processes

Table S 6: Electronic energies (E_e), zero point energies (E_0), enthalpy (H), Gibbs free energies (G), thermal corrections (TC) for T = 363.15 K, and entropy (S) in Hartree obtained at the SMD-(1,4-dioxane)-wB97X-D/aug-cc-pVTZ//SMD-(1,4-dioxane)-M062X/6-31G(d) level of theory

**T=363.15K (90 °C)**	E_e	E_0	$H_{363.15}$	$G_{363.15}$	$G_{363.15}$ **(1M)**	**TC**	**S**	**TS**
**Z-group substituent = *p*-H**								
adduct	-1719.6592	-1719.305	-1719.2719	-1719.3767	-1719.3728	0.386164	757.965	0.104839
fragment A	-1412.5967	-1412.3566	-1412.3321	-1412.4155	-1412.4116	0.263493	603.19	0.083431
fragment B	-1373.2419	-1373.0267	-1373.0064	-1373.0817	-1373.0778	0.234377	544.69	0.07534
dioxane radical	-307.01938	-306.90859	-306.89994	-306.9456	-306.9417	0.118291	330.125	0.045662
MMA radical	-346.39176	-346.25662	-346.24325	-346.3013	-346.2974	0.147358	419.704	0.058052
**Z-group substituent = *p*-OtBu**								
adduct	-1952.1503	-1951.6783	-1951.6358	-1951.7623	-1951.7584	0.51338	914.435	0.126481
fragment A	-1645.0882	-1644.7303	-1644.6964	-1644.8016	-1644.7977	0.390692	760.99	0.105257
fragment B	-1605.7335	-1605.4005	-1605.3708	-1605.4675	-1605.4636	0.361611	699.225	0.096714
**Z-group substituent = *p*-OMe**								
adduct	-1834.1929	-1833.8055	-1833.7688	-1833.8816	-1833.8777	0.423	815.608	0.112812
fragment A	-1527.1314	-1526.8577	-1526.8298	-1526.9211	-1526.9172	0.300462	659.926	0.091279
fragment B	-1487.7769	-1487.5282	-1487.5044	-1487.5875	-1487.5836	0.27133	600.952	0.083122
**Z-group substituent = *p*-OCF_3**								
adduct	-2131.9751	-2131.6114	-2131.5721	-2131.6938	-2131.6899	0.401861	880.235	0.121751
fragment A	-1824.9116	-1824.6615	-1824.631	-1824.7323	-1824.7284	0.279434	732.179	0.101272
fragment B	-1785.5565	-1785.3314	-1785.3051	-1785.3966	-1785.3927	0.250286	661.821	0.091541
**Z-group substituent = *p*-bis-*m*-CF_3**								
adduct	-2393.8401	-2393.4754	-2393.4325	-2393.5611	-2393.5572	0.406468	929.658	0.128587
fragment A	-	-	-	-	-	0.283551	798.492	0.110445

	2086.7714	2086.5213	2086.4867	2086.5972	2086.5933			
fragment B	-2047.4163	-2047.1908	-2047.1605	-2047.2628	-2047.2589	0.254596	739.207	0.102245

Table S 7: Electronic energies (E_e), zero point energies (E_0), enthalpy (H), Gibbs free energies (G), thermal corrections (TC) for T = 373.15 K, and entropy (S) in Hartree obtained at the SMD-(1,4-dioxane)-wB97X-D/aug-cc-pVTZ//SMD-(1,4-dioxane)-M062X/6-31G(d) level of theory

**T=373.15K (100 °C)**	E_e	E_0	$H_{373.15}$	$G_{373.15}$	$G_{373.15}$ **(1M)**	**TC**	**S**	**TS**
**Z-group substituent =** ***p*****-H**								
adduct	-1719.6592	-1719.305	-1719.2702	-1719.3796	-1719.3756	0.387768	769.627	0.109383
fragment A	-1412.5967	-1412.3566	-1412.3309	-1412.4178	-1412.4138	0.264639	611.593	0.086923
fragment B	-1373.2419	-1373.0267	-1373.0054	-1373.0838	-1373.0798	0.235358	551.915	0.078441
dioxane radical	-307.01938	-306.90859	-306.89952	-306.94686	-306.94282	0.118678	333.11	0.047343
MMA radical	-346.39176	-346.25662	-346.24267	-346.30291	-346.29886	0.147904	423.818	0.060235
**Z-group substituent =** ***p*****-OtBu**								
adduct	-1952.1503	-1951.6783	-1951.6337	-1951.7658	-1951.7618	0.515459	929.487	0.132104
fragment A	-1645.0882	-1644.7303	-1644.6947	-1644.8045	-1644.8005	0.392315	772.786	0.109832
fragment B	-1605.7335	-1605.4005	-1605.3693	-1605.4702	-1605.4661	0.363068	709.845	0.100887
**Z-group substituent =** ***p*****-OMe**								
adduct	-1834.1929	-1833.8055	-1833.767	-1833.8847	-1833.8807	0.424766	828.427	0.11774
fragment A	-1527.1314	-1526.8577	-1526.8285	-1526.9236	-1526.9196	0.301769	669.479	0.09515
fragment B	-1487.7769	-1487.5282	-1487.5032	-1487.5898	-1487.5858	0.272474	609.333	0.086602
**Z-group substituent =** ***p*****-OCF$_3$**								
adduct	-2131.9751	-2131.6114	-2131.5702	-2131.6972	-2131.6932	0.403739	893.858	0.12704
fragment A	-1824.9116	-1824.6615	-1824.6296	-1824.7351	-1824.7311	0.280855	742.536	0.105533
fragment B	-1785.5565	-1785.3314	-1785.3038	-1785.3991	-1785.3951	0.251542	671.005	0.095367
**Z-group substituent =** ***p*****-bis-*****m*****-CF$_3$**								
adduct	-2393.8401	-2393.4754	-2393.4304	-2393.5646	-2393.5606	0.408507	944.424	0.134227
fragment A	-2086.7714	-2086.5213	-2086.4851	-2086.6002	-2086.5962	0.285136	810.02	0.115124
fragment B	-2047.4163	-2047.1908	-2047.1591	-2047.2656	-2047.2616	0.256014	749.545	0.106529

Table S 8: Electronic energies (E_e), zero point energies (E_0), enthalpy (H), Gibbs free energies (G), thermal corrections (TC) for T = 393.15 K, and entropy (S) in Hartree obtained at the SMD-(1,4-dioxane)-wB97X-D/aug-cc-pVTZ//SMD-(1,4-dioxane)-M062X/6-31G(d) level of theory

T=393.15K (120 °C)	E_e	E_0	$H_{393.15}$	$G_{393.15}$	$G_{393.15}$ (1M)	TC	S	TS
**Z-group substituent = *p*-H**								
adduct	-1719.6592	-1719.305	-1719.2668	-1719.3856	-1719.3812	0.391083	792.779	0.118713
fragment A	-1412.5967	-1412.3566	-1412.3285	-1412.4226	-1412.4182	0.267006	628.244	0.094075
fragment B	-1373.2419	-1373.0267	-1373.0033	-1373.0881	-1373.0837	0.237393	566.293	0.084798
dioxane radical	-307.01938	-306.90859	-306.89865	-306.94942	-306.9451	0.119484	339.068	0.050773
MMA radical	-346.39176	-346.25662	-346.24148	-346.30617	-346.30184	0.149027	431.953	0.064682
**Z-group substituent = *p*-OtBu**								
adduct	-1952.1503	-1951.6783	-1951.6293	-1951.773	-1951.7687	0.519756	959.364	0.143658
fragment A	-1645.0882	-1644.7303	-1644.6913	-1644.8105	-1644.8062	0.395663	796.167	0.11922
fragment B	-1605.7335	-1605.4005	-1605.3662	-1605.4757	-1605.4713	0.366085	730.957	0.109456
**Z-group substituent = *p*-OMe**								
adduct	-1834.1929	-1833.8055	-1833.7633	-1833.8911	-1833.8868	0.428414	853.859	0.127859
fragment A	-1527.1314	-1526.8577	-1526.8257	-1526.9288	-1526.9245	0.304467	688.397	0.103083
fragment B	-1487.7769	-1487.5282	-1487.5008	-1487.5945	-1487.5902	0.274841	625.99	0.093738
**Z-group substituent = *p*-OCF_3**								
adduct	-2131.9751	-2131.6114	-2131.5662	-2131.7041	-2131.6998	0.407616	920.855	0.137892
fragment A	-1824.9116	-1824.6615	-1824.6266	-1824.7408	-1824.7365	0.283781	763.02	0.114257
fragment B	-1785.5565	-1785.3314	-1785.3011	-1785.4043	-1785.4	0.254137	689.223	0.103206
**Z-group substituent = *p*-bis-*m*-CF_3**								
adduct	-2393.8401	-2393.4754	-2393.4262	-2393.572	-2393.5676	0.412709	973.652	0.145797
fragment A	-2086.7714	-2086.5213	-2086.4818	-2086.6065	-2086.6022	0.288394	832.783	0.124703
fragment B	-2047.4163	-2047.1908	-2047.1561	-2047.2714	-2047.2671	0.258937	770.012	0.115304

4. Cartesian coordinates

Cartesian coordinates (in Å) for all structures optimized at the SMD-(1,4-dioxane)-M062X/6-31G(d) level of theory along with their charges and multiplicities. The number of imaginary frequencies is zero for all structures.

4.1 Structures used to evaluated addition-fragmentation reactions

dioxane radical

0 2

C	1.370763	-0.048561	-0.200873
C	-0.544815	1.274829	-0.156609
C	-1.398102	0.076299	0.112446
C	0.516176	-1.213476	0.259272
H	1.476810	-0.079324	-1.292599
H	2.360148	-0.069897	0.261686
H	-1.623421	-0.006976	1.195037
H	-2.349343	0.158684	-0.420211
H	0.446180	-1.214831	1.359211
H	0.953816	-2.160846	-0.066625
H	-0.950125	2.267574	0.013067
O	0.775795	1.195033	0.170824
O	-0.773070	-1.123648	-0.312697

MMA radical

0 2

C	-0.276807	-0.364326	-0.000168
C	1.091712	0.119396	-0.000412
C	2.200364	-0.871005	0.000226
C	1.378101	1.583128	-0.000072
H	2.454858	1.770526	-0.004505
H	0.943989	2.073937	0.879735
H	0.935971	2.076082	-0.874542
O	-0.608769	-1.538591	-0.000109
O	-1.178866	0.645649	0.000025
C	-2.539938	0.228061	0.000137
H	-3.133176	1.142460	-0.000591
H	-2.763536	-0.366544	0.889642
H	-2.763306	-0.367883	-0.888512
H	1.818000	-1.892548	0.000546
H	2.844038	-0.732360	-0.879008
H	2.843647	-0.731664	0.879639

Z-group substituent = *p*-H

adduct

0 2

C	-3.751638	-1.856545	-0.099460
C	-1.530137	-1.179151	-0.010755
C	-1.155144	-2.490693	0.665362
C	-3.379069	-3.159386	0.584655
H	-4.044199	-1.113088	0.657295
H	-4.574285	-1.995059	-0.805398
H	-0.870121	-3.224419	-0.105277
H	-0.318513	-2.346973	1.355042
H	-3.158659	-3.925255	-0.174897
H	-4.195090	-3.512863	1.220458
H	-1.736372	-0.404018	0.739141

O	-2.646974	-1.365559	-0.851718
O	-2.252116	-2.965988	1.421209
S	-0.199740	-0.594203	-1.128315
C	1.026355	-0.104708	0.032702
S	0.602694	1.047370	1.288553
C	0.198446	2.598871	0.352181
C	-1.049843	2.452409	-0.517576
O	-1.120535	2.840317	-1.657619
C	1.379303	3.019241	-0.511698
H	2.261222	3.179286	0.114698
H	1.611839	2.259433	-1.263480
H	1.142253	3.948973	-1.036577
C	-0.102920	3.643008	1.435926
H	-0.376988	4.590259	0.959258
H	-0.927859	3.326541	2.079185
H	0.784317	3.813840	2.052662
O	-2.086471	1.933906	0.153067
C	-3.280890	1.776002	-0.617206
H	-3.109119	1.077939	-1.439292
H	-4.025155	1.376722	0.071990
H	-3.610559	2.739938	-1.011545
C	2.365569	-0.641358	-0.060016
C	2.636549	-1.826421	-0.786099
C	3.458131	-0.000831	0.572857
C	3.923662	-2.332342	-0.873461
H	1.819946	-2.352021	-1.268657
C	4.740717	-0.516481	0.479528
H	3.284490	0.913147	1.129602
C	4.986341	-1.684071	-0.243736
H	4.099349	-3.246470	-1.432566
H	5.559565	0.000705	0.970554
H	5.992858	-2.083967	-0.314738

fragment A

0 1

S	0.035920	-1.427437	-1.200291
C	-0.604050	-0.135827	-0.414991
S	0.275693	1.269720	0.131579
C	2.044076	0.999421	-0.286871
C	2.577230	-0.362286	0.163149
O	3.471589	-0.935816	-0.406441
C	2.796598	2.058039	0.540183
H	2.463517	3.064682	0.269651
H	2.647076	1.911387	1.613123
H	3.865710	1.981373	0.318722
C	2.297204	1.212115	-1.775947
H	3.360858	1.067615	-1.982628
H	1.732764	0.501340	-2.382618
H	2.014512	2.229945	-2.057535
O	2.042295	-0.769836	1.317170
C	2.520178	-2.032372	1.784734
H	1.969613	-2.236660	2.702051
H	2.322016	-2.807820	1.040744
H	3.593135	-1.986678	1.984465
C	-2.053678	-0.033540	-0.094062

C	-2.996603	-0.515031	-1.011836
C	-2.498422	0.525928	1.111749
C	-4.354010	-0.426012	-0.734568
H	-2.652100	-0.946152	-1.945994
C	-3.858945	0.597735	1.390998
H	-1.780147	0.872692	1.847899
C	-4.789164	0.128519	0.468037
H	-5.074926	-0.790819	-1.459393
H	-4.190445	1.016721	2.335794
H	-5.850948	0.190908	0.685834

fragment B

0 1

C	3.533766	0.696525	1.089947
C	1.913438	-0.140578	-0.338326
C	2.930110	-1.092269	-0.963950
C	4.560188	-0.232434	0.468380
H	3.532678	1.664819	0.568116
H	3.734747	0.861868	2.151109
H	2.908655	-2.058634	-0.435360
H	2.716841	-1.253564	-2.023647
H	4.607980	-1.168486	1.045722
H	5.551386	0.227972	0.452826
H	1.887333	0.800609	-0.905261
O	2.241494	0.104216	1.007383
O	4.214877	-0.511803	-0.879196
S	0.264810	-0.882355	-0.407512
C	-0.797773	0.495122	-0.122103
S	-0.317862	2.056189	-0.021995
C	-2.218338	0.075330	-0.004865
C	-2.575660	-1.119073	0.636176
C	-3.224812	0.890685	-0.540185
C	-3.913476	-1.484333	0.741645
H	-1.811321	-1.744823	1.085913
C	-4.556943	0.512251	-0.447036
H	-2.946907	1.812966	-1.039437
C	-4.905406	-0.674786	0.195942
H	-4.179136	-2.401918	1.256919
H	-5.326350	1.145310	-0.877620
H	-5.948600	-0.965389	0.273572

Z-group substituent = *p*-OtBu

adduct

0 2

C	4.613632	-2.556002	0.348447
C	2.607052	-1.423624	0.039340
C	2.053257	-2.613026	-0.732624
C	4.064344	-3.737147	-0.430639
H	5.141951	-1.875887	-0.336589
H	5.299365	-2.878727	1.135812
H	1.532988	-3.285772	-0.032582
H	1.353087	-2.283288	-1.505360

H	3.600112	-4.453578	0.264532
H	4.859793	-4.242662	-0.984525
H	3.058237	-0.694999	-0.647503
O	3.552343	-1.858449	0.992081
O	3.111140	-3.293603	-1.380070
S	1.304129	-0.591461	1.023967
C	0.354022	0.169618	-0.246008
S	1.154323	1.241705	-1.384212
C	1.751020	2.645925	-0.327100
C	2.837138	2.217621	0.659492
O	2.857907	2.551829	1.818520
C	0.588838	3.272403	0.430621
H	-0.166958	3.629326	-0.274480
H	0.125849	2.553183	1.112434
H	0.946392	4.116914	1.026469
C	2.377862	3.641385	-1.312883
H	2.783506	4.494077	-0.757891
H	3.188225	3.182793	-1.885201
H	1.618749	4.013868	-2.006961
O	3.813617	1.511169	0.075897
C	4.854744	1.084215	0.958113
H	4.451115	0.412272	1.718582
H	5.575670	0.559807	0.330743
H	5.327074	1.945338	1.436469
C	-1.064052	-0.078316	-0.333994
C	-1.663206	-1.191316	0.306942
C	-1.919046	0.776678	-1.073076
C	-3.025007	-1.421173	0.227537
H	-1.038271	-1.886957	0.856008
C	-3.279983	0.542213	-1.150958
H	-1.496152	1.634935	-1.582953
C	-3.851850	-0.551036	-0.491143
H	-3.466392	-2.290363	0.705049
H	-3.921414	1.200893	-1.728068
O	-5.185778	-0.814761	-0.621970
C	-6.104092	-0.258354	0.357742
C	-6.050439	1.266033	0.332705
H	-5.073215	1.638471	0.655626
H	-6.254679	1.641208	-0.675291
H	-6.806451	1.674623	1.010753
C	-7.462906	-0.758056	-0.111160
H	-8.252945	-0.398817	0.554969
H	-7.669267	-0.400671	-1.124328
H	-7.482298	-1.851855	-0.119740
C	-5.785246	-0.788877	1.752081
H	-5.796111	-1.883573	1.758522
H	-4.806045	-0.444707	2.099293
H	-6.537471	-0.434217	2.463860

fragment A

0 1

S	1.918314	1.275776	-1.442046
C	1.070356	0.127615	-0.628708
S	1.766264	-1.115227	0.385657
C	3.580367	-1.080931	0.104891

C	4.208666	0.293786	0.344766
O	5.200281	0.670598	-0.228196
C	3.937525	-1.603671	-1.282350
H	3.585559	-2.632877	-1.391334
H	3.497071	-0.988477	-2.069631
H	5.023588	-1.583524	-1.404296
C	4.149377	-2.012307	1.191389
H	5.237646	-2.053706	1.082805
H	3.909786	-1.652661	2.195620
H	3.755326	-3.026221	1.073605
O	3.626590	0.957020	1.347463
C	4.187936	2.241675	1.619099
H	4.121957	2.875350	0.731175
H	3.592888	2.660021	2.429855
H	5.233652	2.148613	1.920969
C	-0.412733	0.066388	-0.637605
C	-1.154250	1.254605	-0.709927
C	-1.101524	-1.154858	-0.593356
C	-2.539531	1.225834	-0.714820
H	-0.628808	2.202782	-0.752571
C	-2.489344	-1.186470	-0.614036
H	-0.550814	-2.089865	-0.583776
C	-3.217198	0.003998	-0.658917
H	-3.114443	2.144357	-0.772235
H	-3.022381	-2.131482	-0.616395
O	-4.579246	-0.025067	-0.738114
C	-5.350160	-0.012698	0.495258
C	-5.070873	1.262383	1.284363
H	-4.033129	1.302657	1.630049
H	-5.272221	2.146278	0.670719
H	-5.719703	1.301238	2.165068
C	-5.039265	-1.248782	1.333340
H	-5.211484	-2.160455	0.752294
H	-4.003531	-1.246171	1.686902
H	-5.692991	-1.273136	2.210956
C	-6.791149	-0.041088	0.007027
H	-7.481518	-0.033375	0.855668
H	-6.996508	0.831445	-0.620028
H	-6.973394	-0.942861	-0.584900

fragment B

0 1

C	-5.649635	-1.261172	-0.651572
C	-3.609806	-0.265805	-0.143068
C	-4.180908	0.230106	1.179859
C	-6.219768	-0.758644	0.661365
H	-5.850341	-0.531182	-1.449371
H	-6.078398	-2.226239	-0.931925
H	-3.978363	-0.520626	1.959928
H	-3.728135	1.182871	1.462851
H	-6.087179	-1.525003	1.440643
H	-7.284473	-0.531664	0.562463
H	-3.708089	0.508149	-0.916792
O	-4.243814	-1.462859	-0.532755
O	-5.571343	0.441269	1.044758

S	-1.849815	-0.720278	0.003090
C	-0.998689	0.778485	-0.321951
S	-1.706789	2.253922	-0.479216
C	0.467052	0.575376	-0.412276
C	1.018161	-0.603427	-0.937874
C	1.335023	1.588732	0.020880
C	2.393933	-0.765924	-1.021400
H	0.370505	-1.384296	-1.323645
C	2.708711	1.422483	-0.048657
H	0.917479	2.505631	0.423089
C	3.247977	0.239395	-0.563087
H	2.820773	-1.661803	-1.460063
H	3.381099	2.206400	0.284205
O	4.598858	0.107454	-0.697178
C	5.352449	-0.504714	0.386573
C	4.895868	-1.942874	0.609405
H	3.863155	-1.987058	0.969090
H	4.971242	-2.518250	-0.318849
H	5.533026	-2.419656	1.360936
C	5.207344	0.315752	1.664111
H	5.510817	1.353287	1.491443
H	4.176943	0.306846	2.033251
H	5.847580	-0.103054	2.446910
C	6.786584	-0.466344	-0.120516
H	7.464323	-0.903755	0.618459
H	6.874191	-1.030514	-1.053683
H	7.094129	0.566147	-0.310288

Z-group substituent = *p*-OMe

adduct

0 2

C	3.571680	2.842873	-0.137894
C	1.673331	1.506988	-0.004969
C	0.933741	2.628065	0.711028
C	2.836151	3.956395	0.585342
H	4.105099	2.217723	0.593967
H	4.287508	3.239969	-0.862014
H	0.410396	3.246859	-0.035113
H	0.206178	2.222651	1.419734
H	2.364420	4.625553	-0.150792
H	3.522763	4.537812	1.206361
H	2.134906	0.824826	0.722118
O	2.650269	2.042942	-0.870973
O	1.852892	3.411983	1.447538
S	0.555657	0.549478	-1.097181
C	-0.433360	-0.293084	0.091403
S	0.355978	-1.301315	1.293446
C	1.167177	-2.636612	0.292496
C	2.301039	-2.104293	-0.583402
O	2.468971	-2.429275	-1.733051
C	0.140704	-3.351112	-0.575290
H	-0.645308	-3.779253	0.052995
H	-0.315937	-2.665544	-1.294944

H	0.624669	-4.154762	-1.137422
C	1.782867	-3.586835	1.328846
H	2.313987	-4.393016	0.811453
H	2.490649	-3.066511	1.979271
H	0.996733	-4.033734	1.944480
O	3.144344	-1.312716	0.091728
C	4.217135	-0.776287	-0.685808
H	3.822836	-0.141025	-1.482089
H	4.818073	-0.187923	0.007950
H	4.817399	-1.581223	-1.116202
C	-1.866300	-0.154320	0.071164
C	-2.503250	0.903565	-0.617859
C	-2.712705	-1.075073	0.745870
C	-3.882721	1.042224	-0.641272
H	-1.896826	1.638962	-1.135024
C	-4.082713	-0.941781	0.726681
H	-2.268372	-1.907314	1.280180
C	-4.687944	0.118210	0.032777
H	-4.319110	1.876193	-1.178548
H	-4.721484	-1.654469	1.238328
O	-6.037942	0.161073	0.074095
C	-6.681720	1.211722	-0.621488
H	-6.459393	1.173844	-1.694179
H	-7.751063	1.063611	-0.469294
H	-6.391216	2.190259	-0.221919

fragment A

0 1

S	-0.773257	1.244250	1.339876
C	-0.095490	0.064564	0.414401
S	-0.991952	-1.131764	-0.497043
C	-2.742158	-1.019838	0.045469
C	-3.338229	0.382305	-0.098176
O	-4.220022	0.796016	0.612717
C	-2.919337	-1.538816	1.468345
H	-2.598244	-2.582311	1.523643
H	-2.346088	-0.948254	2.185838
H	-3.975211	-1.475594	1.744090
C	-3.502713	-1.916391	-0.949480
H	-4.564944	-1.910404	-0.685830
H	-3.393752	-1.559572	-1.977142
H	-3.141491	-2.947511	-0.891082
O	-2.879492	1.028583	-1.173302
C	-3.416155	2.339021	-1.355628
H	-2.925765	2.739994	-2.241722
H	-4.497499	2.294766	-1.504244
H	-3.194958	2.959475	-0.483592
C	1.365607	-0.066670	0.218384
C	2.176353	1.073741	0.256572
C	1.983717	-1.311302	-0.004967
C	3.549279	0.995815	0.069701
H	1.714326	2.041439	0.421456
C	3.350960	-1.404088	-0.178188
H	1.392195	-2.221039	-0.007385
C	4.146162	-0.250963	-0.147662

H	4.140064	1.903694	0.091204
H	3.834289	-2.363174	-0.330584
O	5.468400	-0.440838	-0.333916
C	6.310878	0.697052	-0.292004
H	6.254062	1.197629	0.681208
H	7.323857	0.326390	-0.448933
H	6.058162	1.408026	-1.086847

fragment B

0 1

C	-4.778009	-1.078799	-0.686311
C	-2.690629	-0.201531	-0.151552
C	-3.319589	0.606699	0.976883
C	-5.403525	-0.267821	0.432573
H	-4.818663	-0.509793	-1.626695
H	-5.288998	-2.034863	-0.822966
H	-3.277898	0.016113	1.905637
H	-2.786907	1.549537	1.118524
H	-5.431901	-0.868582	1.354767
H	-6.421439	0.034417	0.172837
H	-2.625273	0.404173	-1.065929
O	-3.422426	-1.386172	-0.373063
O	-4.658242	0.917613	0.647641
S	-1.017619	-0.786008	0.278361
C	0.034563	0.520079	-0.242190
S	-0.486239	1.997649	-0.748536
C	1.463730	0.156716	-0.146538
C	1.913433	-1.166246	-0.320156
C	2.416872	1.147201	0.119525
C	3.256380	-1.475939	-0.235797
H	1.209277	-1.955952	-0.561627
C	3.767692	0.846439	0.221412
H	2.086058	2.170245	0.264349
C	4.195879	-0.473446	0.040581
H	3.609732	-2.489958	-0.388941
H	4.472458	1.638458	0.444371
O	5.481217	-0.873227	0.111277
C	6.464962	0.111117	0.376562
H	6.300924	0.583052	1.351965
H	7.420134	-0.413861	0.385459
H	6.479272	0.878231	-0.405732

Z-group substituent = *p*-OCF_3

adduct

0 2

C	4.595003	-2.535570	0.356303
C	2.583359	-1.413120	0.040878
C	2.036587	-2.606890	-0.729934
C	4.052304	-3.720539	-0.421180
H	5.122004	-1.854648	-0.328728
H	5.279416	-2.852975	1.146773
H	1.518631	-3.281041	-0.029491

H	1.336204	-2.282137	-1.504725
H	3.589105	-4.437566	0.273864
H	4.850734	-4.223668	-0.972710
H	3.029250	-0.681333	-0.645725
O	3.528565	-1.840362	0.995633
O	3.099235	-3.281841	-1.373669
S	1.272275	-0.592504	1.027099
C	0.319849	0.167082	-0.238445
S	1.109229	1.240727	-1.381084
C	1.715901	2.643047	-0.325063
C	2.812759	2.214099	0.649413
O	2.841411	2.542192	1.809692
C	0.559846	3.265609	0.445123
H	-0.203856	3.622632	-0.251314
H	0.105547	2.546048	1.132394
H	0.922423	4.110372	1.037425
C	2.330138	3.640786	-1.316484
H	2.739739	4.492885	-0.763631
H	3.135591	3.185165	-1.897937
H	1.563042	4.013605	-2.001500
O	3.786722	1.515262	0.053090
C	4.843843	1.097475	0.921586
H	4.457287	0.418471	1.684617
H	5.563391	0.584521	0.283425
H	5.311262	1.962763	1.397007
C	-1.101263	-0.083902	-0.321013
C	-1.689623	-1.202252	0.318798
C	-1.957004	0.779597	-1.047594
C	-3.051207	-1.440245	0.242238
H	-1.061077	-1.893467	0.868390
C	-3.318345	0.542516	-1.128063
H	-1.538583	1.648062	-1.542674
C	-3.852899	-0.565792	-0.481589
H	-3.498811	-2.302028	0.725818
H	-3.972588	1.207136	-1.682167
O	-5.218568	-0.835629	-0.603393
C	-6.006444	-0.273863	0.337153
F	-5.933363	1.060891	0.326015
F	-7.257362	-0.629628	0.088991
F	-5.685776	-0.675659	1.570667

fragment A

0 1

S	-1.915158	-1.405600	-1.288641
C	-1.054677	-0.289889	-0.446292
S	-1.721940	1.078337	0.409679
C	-3.489593	1.199069	-0.072892
C	-4.275117	-0.092569	0.166875
O	-5.231587	-0.409533	-0.494808
C	-3.638861	1.662626	-1.517801
H	-3.171513	2.642978	-1.641778
H	-3.184134	0.957110	-2.216333
H	-4.701746	1.741517	-1.760298
C	-4.073557	2.248253	0.891077
H	-5.133419	2.387800	0.656560

H	-3.983064	1.931667	1.933565
H	-3.566393	3.209680	0.765952
O	-3.872238	-0.743458	1.261116
C	-4.586459	-1.949758	1.535215
H	-4.493661	-2.640202	0.693206
H	-4.124019	-2.372141	2.426279
H	-5.642695	-1.738123	1.715985
C	0.421035	-0.373361	-0.289772
C	1.038131	-1.628831	-0.191484
C	1.224196	0.771792	-0.250623
C	2.409596	-1.731702	-0.046648
H	0.427698	-2.524872	-0.217008
C	2.606271	0.683915	-0.121966
H	0.777116	1.754538	-0.356851
C	3.186121	-0.575682	-0.015587
H	2.897117	-2.696056	0.044898
H	3.199329	1.589266	-0.114937
O	4.544272	-0.807381	0.121303
C	5.404400	0.231505	0.195805
F	5.144933	1.030433	1.233854
F	6.619914	-0.267279	0.340907
F	5.384094	0.986160	-0.906114

fragment B

0 1

C	-5.302487	0.629878	-1.094196
C	-3.673059	-0.168796	0.345607
C	-4.667277	-1.153592	0.956062
C	-6.307482	-0.331469	-0.487163
H	-5.333844	1.595170	-0.567868
H	-5.497454	0.794567	-2.156530
H	-4.613823	-2.115680	0.421985
H	-4.459655	-1.315204	2.016827
H	-6.322983	-1.265241	-1.069841
H	-7.311540	0.100283	-0.478291
H	-3.680161	0.769053	0.918508
O	-3.994433	0.074034	-1.001603
O	-5.966455	-0.608146	0.862200
S	-2.003136	-0.861534	0.424719
C	-0.982727	0.546672	0.134108
S	-1.508822	2.093445	0.045199
C	0.446879	0.170687	0.000437
C	0.840480	-1.027297	-0.613669
C	1.437873	1.029867	0.488900
C	2.182805	-1.348266	-0.737292
H	0.098537	-1.698504	-1.033318
C	2.785342	0.713543	0.388296
H	1.143859	1.956961	0.968979
C	3.145337	-0.481378	-0.231566
H	2.500519	-2.261318	-1.228530
H	3.524773	1.393971	0.790831
O	4.448531	-0.916134	-0.402258
C	5.479798	-0.137641	-0.007543
F	5.478874	1.050705	-0.615391
F	5.470759	0.084744	1.309601

F	6.598041	-0.770552	-0.318658

Z-group substituent = *p*-bis-*m*-CF_3

adduct

0 2

C	4.505661	0.245367	-0.055705
C	2.862598	-1.288349	-0.665485
C	3.880896	-2.048608	-1.511115
C	5.521496	-0.508466	-0.891841
H	4.028862	1.033996	-0.655296
H	4.962980	0.696059	0.827844
H	4.330959	-2.851319	-0.904684
H	3.402708	-2.486932	-2.389920
H	6.041287	-1.248015	-0.263889
H	6.258237	0.172868	-1.324710
H	2.344062	-0.546426	-1.289837
O	3.513162	-0.663014	0.418393
O	4.873978	-1.157946	-1.973564
S	1.617240	-2.457579	-0.016155
C	0.093084	-1.655334	-0.298481
S	-1.220066	-2.729741	-0.723228
C	-2.303615	-2.696079	0.792873
C	-2.976112	-1.334843	0.955306
O	-2.826123	-0.618193	1.914678
C	-1.484221	-3.012008	2.035487
H	-0.983131	-3.976895	1.918652
H	-0.735049	-2.239305	2.226596
H	-2.143076	-3.058140	2.908288
C	-3.368783	-3.766076	0.531662
H	-4.086831	-3.772993	1.358716
H	-3.914503	-3.575482	-0.395224
H	-2.901090	-4.753107	0.475106
O	-3.792243	-1.042876	-0.065321
C	-4.514220	0.184342	0.069775
H	-3.837636	1.009380	0.297560
H	-5.013844	0.344998	-0.884101
H	-5.248912	0.097834	0.874599
C	-0.092001	-0.226866	-0.159037
C	0.745957	0.544139	0.667149
C	-1.111719	0.441945	-0.875581
C	0.584954	1.922283	0.747883
H	1.528330	0.064062	1.246411
C	-1.252561	1.813633	-0.773105
H	-1.771870	-0.125043	-1.523872
C	-0.407479	2.579098	0.034933
H	-0.526393	3.655370	0.101243
C	1.540939	2.682621	1.622544
C	-2.317849	2.530490	-1.552660
F	-3.059893	1.695009	-2.291984
F	-1.792168	3.443534	-2.382398
F	-3.157137	3.193268	-0.737561
F	1.255314	3.989337	1.672854
F	1.539701	2.218303	2.879094

F	2.805053	2.568184	1.173943

fragment A

0 1

S	1.655313	-1.882411	0.696538
C	1.035736	-0.596801	-0.107946
S	1.917409	0.539706	-1.089648
C	3.689270	0.058014	-1.008653
C	4.194064	-0.160249	0.419832
O	5.090052	-0.920731	0.686182
C	4.442287	1.284353	-1.556229
H	4.130630	1.500894	-2.582481
H	4.272245	2.170182	-0.938712
H	5.513475	1.060345	-1.565509
C	3.964586	-1.166721	-1.874921
H	5.028670	-1.410340	-1.819293
H	3.399015	-2.036051	-1.533759
H	3.702486	-0.951532	-2.914174
O	3.629435	0.671026	1.298946
C	4.086659	0.519227	2.645018
H	3.516690	1.236287	3.234022
H	3.896221	-0.498674	2.993698
H	5.155786	0.731811	2.712286
C	-0.421399	-0.274502	-0.071239
C	-1.355455	-1.317115	-0.088808
C	-0.875738	1.044724	-0.002077
C	-2.712486	-1.033998	-0.050836
H	-1.010282	-2.344292	-0.143753
C	-2.241885	1.308706	0.050260
H	-0.170811	1.869589	0.043564
C	-3.171353	0.279334	0.021135
H	-4.234519	0.491892	0.054187
C	-3.707835	-2.161328	-0.031970
C	-2.693620	2.742050	0.094131
F	-1.954603	3.460954	0.950854
F	-3.974016	2.852956	0.467446
F	-2.575720	3.328897	-1.106400
F	-4.857554	-1.810523	-0.625168
F	-4.010790	-2.528491	1.221690
F	-3.240209	-3.247498	-0.659257

fragment B

0 1

C	5.279437	-0.305208	-1.246212
C	3.649861	-0.209594	0.397569
C	4.644754	0.407785	1.377460
C	6.284508	0.293221	-0.279803
H	5.308472	-1.403371	-1.194625
H	5.473952	0.010684	-2.273754
H	4.593759	1.506421	1.315757
H	4.435099	0.091769	2.402533
H	6.301913	1.387520	-0.395599
H	7.287907	-0.101021	-0.459226
H	3.649671	-1.303387	0.506820

O	3.971070	0.156530	-0.920065
O	5.941375	-0.047065	1.054764
S	1.981488	0.390061	0.767732
C	0.964306	-0.737456	-0.117173
S	1.468732	-2.081775	-0.894750
C	-0.470786	-0.337391	-0.066415
C	-0.855773	1.002934	-0.155983
C	-1.455891	-1.323771	0.061106
C	-2.205441	1.342380	-0.121794
H	-0.112062	1.782873	-0.287525
C	-2.794358	-0.964717	0.110220
H	-1.163776	-2.366078	0.133924
C	-3.184789	0.369121	0.017564
H	-4.234722	0.640407	0.053027
C	-3.849650	-2.031049	0.213336
C	-2.589513	2.794779	-0.185396
F	-4.916835	-1.598214	0.899053
F	-4.288602	-2.407700	-0.996415
F	-3.388605	-3.128363	0.825879
F	-1.805812	3.472592	-1.034742
F	-2.469208	3.386355	1.012637
F	-3.857970	2.957518	-0.581245

5. References

1. M. L. Allegrezza, N. D. A. Watuthanthrige, Y. Wang, G. A. Garcia, H. Ren and D. Konkolewicz, *Polym. Chem.*, 2020, **11**, 6129-6133.
2. M. Benaglia, E. Rizzardo, A. Alberti and M. Guerra, *Macromolecules*, 2005, **38**, 3129-3140.
3. Y. Chong, J. Krstina, T. P. Le, G. Moad, A. Postma, E. Rizzardo and S. H. Thang, *Macromolecules*, 2003, **36**, 2256-2272.
4. C. Hansch, A. Leo and R. Taft, *Chem. Rev.*, 1991, **91**, 165-195.
5. P. Ertl, *Chemistry-Methods*, 2022, **2**, e202200041.
6. A. V. Marenich, C. J. Cramer and D. G. Truhlar, *J. Phys. Chem. B*, 2009, **113**, 6378-6396.
7. Y. Zhao and D. G. Truhlar, *Theor. Chem. Acc.*, 2008, **120**, 215-241.
8. W. J. Hehre, R. Ditchfield and J. A. Pople, *J. Chem. Phys.*, 1972, **56**, 2257-2261.
9. P. C. Hariharan and J. A. Pople, *Theoretica chimica acta*, 1973, **28**, 213-222.
10. M. M. Francl, W. J. Pietro, W. J. Hehre, J. S. Binkley, M. S. Gordon, D. J. DeFrees and J. A. Pople, *J. Chem. Phys.*, 1982, **77**, 3654-3665.
11. M. S. Gordon, J. S. Binkley, J. A. Pople, W. J. Pietro and W. J. Hehre, *J. Am. Chem. Soc.*, 1982, **104**, 2797-2803.
12. M. L. Coote, E. H. Krenske and E. I. Izgorodina, *Macromol. Rapid Commun.*, 2006, **27**, 473-497.
13. J.-D. Chai and M. Head-Gordon, *Physical Chemistry Chemical Physics*, 2008, **10**, 6615-6620.
14. R. A. Kendall, T. H. Dunning, Jr. and R. J. Harrison, *J. Chem. Phys.*, 1992, **96**, 6796-6806.
15. D. E. Woon and T. H. Dunning, Jr., *J. Chem. Phys.*, 1993, **98**, 1358-1371.

16. R. F. Ribeiro, A. V. Marenich, C. J. Cramer and D. G. Truhlar, *J. Phys. Chem. B*, 2011, **115**, 14556-14562.
17. J. Ho, A. Klamt and M. L. Coote, *J. Phys. Chem. A*, 2010, **114**, 13442-13444.
18. M. Frisch, G. Trucks, H. Schlegel, G. Scuseria, M. Robb, J. Cheeseman, G. Scalmani, V. Barone, G. Petersson and H. Nakatsuji, *Gaussian Inc. Wallingford CT*, 2016, **1**, 572.
19. C. Y. Legault, CYLview20, http://www.cylview.org
20. S. Perrier, *Macromolecules*, 2017, **50**, 7433-7447.
